# Contribution of genetic and epigenetic changes to escape from X-chromosome inactivation

**DOI:** 10.1186/s13072-021-00404-9

**Published:** 2021-06-29

**Authors:** Bradley P. Balaton, Carolyn J. Brown

**Affiliations:** Department of Medical Genetics, The University of British Columbia, Vancouver, Canada

**Keywords:** X-chromosome inactivation, Epigenetics, DNA methylation, Histone marks, H3K27me3, H3K4me3, H3K9me3, Inactivation status, Escape

## Abstract

**Background:**

X-chromosome inactivation (XCI) is the epigenetic inactivation of one of two X chromosomes in XX eutherian mammals. The inactive X chromosome is the result of multiple silencing pathways that act in concert to deposit chromatin changes, including DNA methylation and histone modifications. Yet over 15% of genes escape or variably escape from inactivation and continue to be expressed from the otherwise inactive X chromosome. To the extent that they have been studied, epigenetic marks correlate with this expression.

**Results:**

Using publicly available data, we compared XCI status calls with DNA methylation, H3K4me1, H3K4me3, H3K9me3, H3K27ac, H3K27me3 and H3K36me3. At genes subject to XCI we found heterochromatic marks enriched, and euchromatic marks depleted on the inactive X when compared to the active X. Genes escaping XCI were more similar between the active and inactive X. Using sample-specific XCI status calls, we found some marks differed significantly with variable XCI status, but which marks were significant was not consistent between genes. A model trained to predict XCI status from these epigenetic marks obtained over 75% accuracy for genes escaping and over 90% for genes subject to XCI. This model made novel XCI status calls for genes without allelic differences or CpG islands required for other methods. Examining these calls across a domain of variably escaping genes, we saw XCI status vary across individual genes rather than at the domain level. Lastly, we compared XCI status calls to genetic polymorphisms, finding multiple loci associated with XCI status changes at variably escaping genes, but none individually sufficient to induce an XCI status change.

**Conclusion:**

The control of expression from the inactive X chromosome is multifaceted, but ultimately regulated at the individual gene level with detectable but limited impact of distant polymorphisms. On the inactive X, at silenced genes euchromatic marks are depleted while heterochromatic marks are enriched. Genes escaping inactivation show a less significant enrichment of heterochromatic marks and depletion of H3K27ac. Combining all examined marks improved XCI status prediction, particularly for genes without CpG islands or polymorphisms, as no single feature is a consistent feature of silenced or expressed genes.

**Supplementary Information:**

The online version contains supplementary material available at 10.1186/s13072-021-00404-9.

## Introduction

In eutherian mammals, one of the two X chromosomes (X) is epigenetically inactivated in XX females in order to achieve dosage compensation with XY males through a process known as X-chromosome inactivation (XCI) (see Balaton, 2018 for a review [1]). This inactivation is incomplete, as approximately 12% of genes consistently escape from XCI in humans [2], here defined as having at least 10% expression from the inactive X (Xi) as compared to the active X (Xa) [3]. There is growing interest in genes that escape XCI for their possible roles in sex-biased disease. Having two active copies of a gene offers additional protection from loss of function mutations linked to cancer [4] and likely underlies other sex-biased diseases and sex chromosome aneuploidies [5]. Genes that escape XCI tend to have sex-biased expression, being higher in males for genes that are also on the Y chromosome while being higher in females if the gene is only on the X [6, 7]. However, relatively little is known about how a gene can be expressed from the midst of heterochromatin.

The pseudoautosomal regions (PAR) are located at the ends of the X and Y chromosomes and maintain their ability to pair and recombine [8]. PAR1 contains approximately 30% of the genes described as escaping from XCI [2]. The short arm of the X near PAR1 is enriched in genes escaping from XCI, while the long arm that contains XIST—the gene responsible for initiating XCI—is enriched in genes subject to XCI [3]. Genes escaping from XCI are often found clustered together, with some convergence with topologically associated domains (TADs) [9]. In addition to genes that consistently escape from XCI (sometimes called constitutive escape), a further 8% of genes have been found to vary their XCI status between different tissues or individuals (termed variable or facultative escape [2] (reviewed in [5]), and another 7% of genes were found to be discordant between the studies identifying them [2]. Variably escaping and discordant genes were found to be enriched at boundaries between clusters of genes with opposite XCI statuses [2]. The factors determining XCI status remain unresolved, with the above evidence suggesting regional control, but there are also lone genes that escape XCI while flanked with genes subject to XCI [2] and even genes with two transcription start sites (TSSs) with opposite XCI status [10, 11]. Furthermore, these solo escape genes are able to recapitulate escape when integrated elsewhere on the X [12, 13].

Many methods have been used to identify which genes escape from XCI (reviewed in [14]). The gold-standard approach is to compare expression levels between the Xi and Xa within a sample [3, 6, 15], which requires a heterozygous SNP within an exon to differentiate Xi from Xa expression. Such expressed SNPs can be rare, and additionally, which X is inactivated is normally random throughout a subject’s body, precluding assigning an allele to the Xi. Some samples are naturally skewed so that the same X is inactivated in > 90% of their cells, which allows allelic analysis. The frequency of such skewing is increased in blood [16], with age [17] and also in cancer, which generally arises clonally [18]. Cells that have become monoclonal during cell culture and those skewed due to deleterious alleles on one copy of the X have also been used for allelic expression analysis [3]. Single-cell RNA-sequencing (RNA-seq) has also been used to avoid the need for clonal cell populations when using Xi/Xa expression to make XCI status calls [19]. Single-cell RNA-seq can additionally see variation in XCI status between different Xi alleles within the same sample and heterogeneity has even been observed in XCI status of a gene in cells with the same Xi [6, 20].

Beyond the direct examination of allelic expression, the modifications to DNA and chromatin that accompany XCI can be used as surrogates to determine if a gene is inactivated. For these features it is unclear if the mark enables or reflects XCI status. Promoter DNA methylation (DNAme) at CpG islands is strongly predictive of a gene’s XCI status and has been used to differentiate genes that escape XCI from those subject to XCI without the need for heterozygous SNPs or skewed Xi choice [21]. Low promoter DNAme on the Xa, as evidenced by low DNAme in male samples, is necessary to allow detection of DNAme differences on the Xi. Genes that escape from XCI will also have low DNAme in females, with both the Xi and Xa unmethylated, while genes that are subject to XCI will have intermediate DNAme, with the Xa unmethylated and the Xi methylated. Other epigenetic marks such as histone marks have been reported to be correlated with a gene’s XCI status. Active marks such as H3K4me2/3, H3K9ac, H3K27ac, H3K9me1, RNA polymerase II and transposase accessibility are enriched at genes escaping from XCI, while inactive marks such as H3K9me3, H4K20me3, H3K27me3 and macroH2A are enriched at genes subject to XCI [14, 22, 23], reviewed in [14]. A predictive model using many epigenetic as well as genetic features in mice was able to predict a gene’s XCI status accurately 78% of the time [24] and in humans a model obtained over 80% accuracy using only genomic repeats [25]. These, and additional studies have found L1 repeats enriched near genes that are subject to XCI, while ALU elements are more frequent at genes escaping XCI [25–28].

Little is known about genetic changes that might predispose genes towards escape from XCI beyond these associations with repetitive elements. Few XCI-related genetic association studies have previously been performed. One study found an association between autosomal variants and DNAme at variably escaping genes [29]. Another study found many genes where Xi expression and in some cases, XCI status, change depending on which allele was on the Xi [6]. Many association studies remove the X from their analysis or fail to account for the effect of male:female copy number differences and differences in expressed copy number due to XCI status. There are various methods which work to avoid these problems using statistical models [30, 31].

In order to understand how genes are (or are not) silenced on the Xi, in a chromosome-wide fashion, we take advantage of the growing genome-wide epigenomic datasets to correlate XCI status with multiple epigenetic marks. The strength of these correlations permitted the development of an epigenetic predictor of XCI status, allowing prediction of escape. Within a single region, we observed control of silencing/escape at the single gene level, while extending our study to additional datasets we observed autosomal variants that often impacted multiple variable escapees, but generally with only minimal effect size.

## Results

To understand the interplay of epigenetic marks and XCI status, we sought a dataset with both a broad range of epigenetic marks and matched expression data to determine XCI status. We thus turned to data from the International Human Epigenome Consortium (IHEC), which has standardized chromatin immunoprecipitation sequencing (ChIP-seq) for the core histone marks H3K4me1, H3K4me3, H3K9me3, H3K27ac, H3K27me3 and H3K36me3 along with whole genome bisulfite sequencing (WGBS) to examine DNAme. Given the heterogeneity in XCI status among tissues and individuals, we focussed on data from the Center for Epigenome Mapping Technologies (CEMT) as these samples were derived from cancer and thus were anticipated to have a high frequency of skewed XCI, allowing us to use allelic expression to determine XCI status in each sample [11]. As cancer is known to have epigenetic changes, we additionally examined data from Core Research for Evolutional Science and Technology (CREST), another group within IHEC, thus allowing us to determine whether any trends that we observed in the CEMT data were due to the samples being cancer-derived. However, the CREST samples had less sequencing depth, fewer females (only nine), and could only be examined for DNAme and histone marks. Samples are listed in Additional file 2: Table S1. In our analyses, genes in the PAR were not included with genes escaping from XCI as they may be epigenetically distinct, especially when comparisons with males are included.

### Histone marks differ with sex and XCI status

**Fig. 1:**
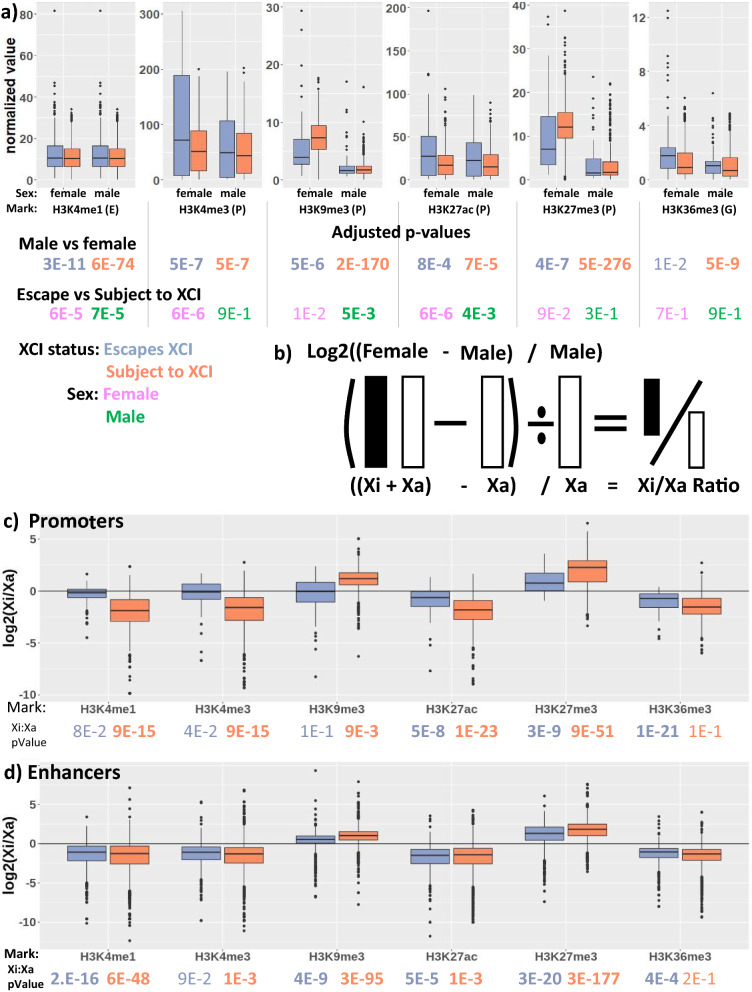
The Xi has more heterochromatic and less euchromatic marks than the Xa. **a** Normalized values for each histone mark, split by sex and XCI status. Each data point is the median normalized ChIP-seq signal across samples for one TSS, enhancer or transcript. All marks noted with (P) had data within 500 bp of the promoter used, while H3K4me1 used enhancers and H3K36me3 used gene body signal. **b** a diagram showing our Xi/Xa calculation. **c**, **d** Log_2_(Xi/Xa ratio) for the histone marks examined here at promoters (**c**) and enhancers (**d**), split by XCI meta-status. Data from CEMT is shown. Underneath, the adjusted p-values for various t-test comparisons are shown, with colors denoting which groups are being compared. See Additional file 3: Table S2 for other comparisons. There are 102 transcripts which escape XCI and 993 transcripts which are subject to XCI. There are 1036 enhancers annotated to genes which escape from XCI and 5077 which are annotated to genes which are subject to XCI

We compared the levels of histone modifications with sex and published XCI status calls derived from a synthesis of various approaches (hereafter referred to as meta-status) [2]. We used levels within 500 bp upstream of a gene’s TSS (except for the mark H3K36me3 that is associated with gene bodies and so was examined at exons [32]), and H3K4me1 that is associated with enhancers and so was examined at annotated enhancer sites [33]. We found that most marks had a significant difference (*p* value < 0.01) for the median level per transcript between males and females, at genes escaping and subject to XCI in both datasets (Fig. 1a, Additional file 3: Table S2). Fewer marks showed significant differences between genes escaping XCI and those subject to XCI within each sex. The euchromatic marks (H3K4me3, H3K27ac, and H3K36me3) were significantly different between transcripts subject to XCI and those escaping from XCI in both CEMT and CREST females, while the heterochromatic marks (H3K9me3, and H3K27me3) were only significantly different within the CREST dataset. Comparing XCI statuses within males gave the fewest significantly different marks, as was expected. Overall, the X chromosome of males and females differs in both heterochromatic and euchromatic marks, and the observable differences between XCI status implicate inactivation-related differences in addition to copy number (XX or XY) differences.

To visualize the chromatin differences between the Xi and Xa, we calculated the Xi to Xa fold change for each mark by taking the female:male difference (the contribution from the Xi) and dividing it by the male value (the contribution from the Xa) (Fig. 1b, c, Additional file 1: Figure S1a for CREST, Additional file 3: Table S2 for values). Heterochromatic marks are generally higher on the Xi than Xa, especially for transcripts subject to XCI. There are almost ten times as many transcripts subject to XCI as there are escaping XCI, which partially explains the stronger p-values at transcripts subject to XCI. H3K27me3 has a higher Xi:Xa fold change than H3K9me3 in both XCI statuses and both marks are highest at transcripts subject to XCI. For euchromatic marks, the Xi:Xa ratio is close to 1:1 at transcripts escaping XCI, and lower for transcripts subject to XCI. H3K36me3 is reduced on the Xi for both XCI statuses with the Xi being approximately one half of the Xa. For transcripts subject to XCI, the healthy CREST samples had less of an Xi to Xa difference than the CEMT cancer samples while at transcripts escaping from XCI the differences were more variable between the datasets.

H3K27me3 showed the largest change between the Xi and Xa, yet was not significantly different between XCI status in females (or males). We examined the proportion of transcripts that showed significant male:female differences, subdivided by XCI meta-status (Additional file 1: Table S3). While DNAme and H3K9me3 show substantial enrichment in females predominantly at transcripts subject to XCI, we found H3K27me3 significant in over 85% of the transcripts in any XCI status. To validate this broad enrichment across the Xi, we examined H3K27me3 data from ENCODE and found a similar trend to the CEMT data, with over 70% of transcripts in the escaping, subject to XCI and variably escaping categories being significantly different between the sexes. We analyzed chromosome 7 as an example autosome and saw a much lower percentage of transcripts with significant male–female differences for H3K9me3 and H3K27me3 than for transcripts escaping from XCI, validating that transcripts that escape from XCI have a significant increase of heterochromatic marks in females relative to males. Metagene plots extending 50 kb up and downstream of genes escaping or subject to XCI, in females and males (Additional file 1:Figure S2) confirm the predominance of marks at the TSSs, with higher H3K4me3 and H3K27ac TSS peaks observed for genes escaping XCI in females. For the heterochromatic H3K9me3 and particularly H3K27me3, we observe both a reduced TSS peak and lower gene body levels for escape genes in females. For all marks, the standard deviation across genes with each XCI status was large, calling into question whether the differences could be predictive for individual genes, as has been found for DNAme (see Additional file 3: Table S2).

In addition to our promoter and gene-based analysis, we also compared histone marks at enhancers annotated to genes on the X [33] and found that all marks showed significant, although small, differences between males and females, for both XCI statuses (Fig. 1d, Additional file 3: Table S2 for values). We further considered whether the enhancer was found within the gene to ensure that differences were not arising simply due to expression of the gene altering chromatin; however, most marks remained significant regardless of location. Looking at the Xi:Xa fold change at enhancers (Fig. 1d, see Additional file 1: Figure S3 for division into genic and intergenic enhancers, Additional file 1: Figure S1b for CREST), all of the heterochromatic marks were higher on the Xi than the Xa, while euchromatic marks were higher on the Xa than the Xi. This did not differ greatly between enhancers that were annotated to interact with genes escaping and genes subject to XCI. In CREST, the Xi and Xa had less differences at enhancers although most were still significantly different at genes subject to XCI, with only heterochromatic marks being significant at genes escaping from XCI. Overall, it appears that enhancers gain heterochromatic marks on the Xi, regardless of XCI status.

### Epigenetic marks correlate with sample-specific changes in XCI status

**Fig. 2:**
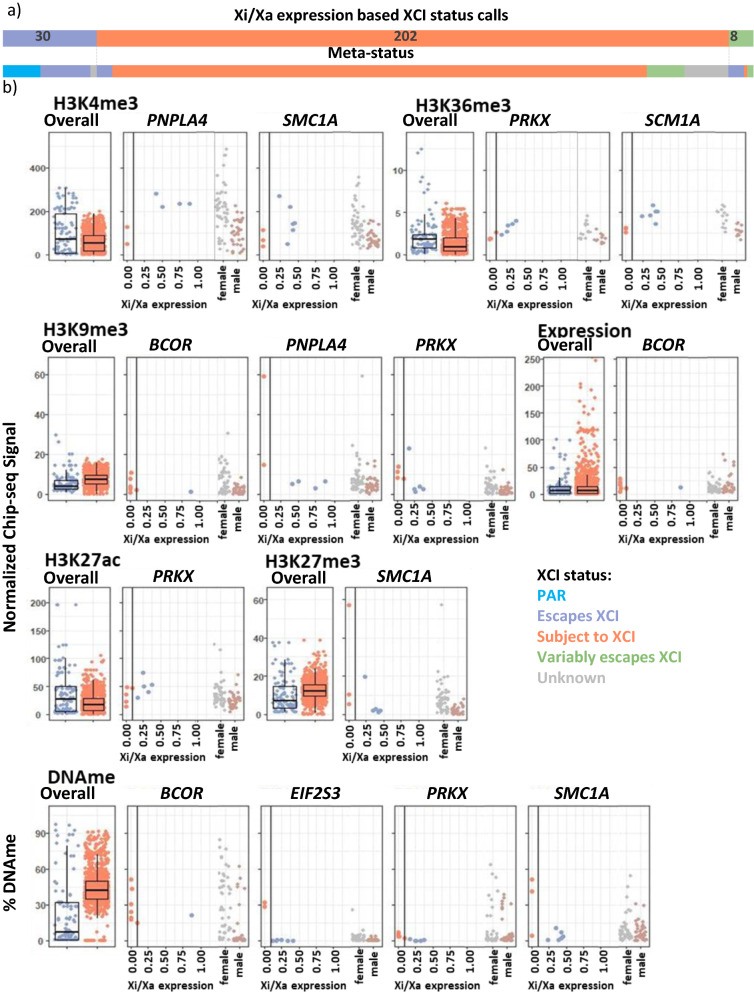
Epigenetic marks do not change consistently with XCI status for variably escaping genes. **a** The number of genes with each XCI status call across all samples as assigned by Xi/Xa expression with their call by meta-status underneath. **b** The correlation of histone marks and Xi/Xa expression determined XCI status. On the left for each mark is a comparison to the overall XCI status across samples per gene, with each data point being a separate gene averaged across all female samples. On the right are shown the variably escaping genes that had significant differences in the histone mark between samples that were subject to or escaping from XCI. For each gene, on the left is a comparison of each epigenetic mark vs the Xi/Xa expression ratio for each informative sample, with allelic expression on the X axis and the epigenetic mark on the Y axis; on the right is the data for all samples, split by sex. A p-value of 0.05 was used for significance. Unknown XCI status is for samples that were uninformative in the Xi/Xa expression analysis. The scale for expression has been reduced, as there are many genes whose expression is much larger than BCOR, making the differences indistinguishable

Heterogeneity of XCI status means that our use of meta-status will be less accurate than individual-specific XCI calls. We thus used our Xi/Xa expression-based XCI status calls in a subset of the CEMT samples with skewed XCI (as reported previously [11]) to analyze the correlation between XCI status and epigenetic marks, made within the same sample. For this sample-specific expression analysis, we could only examine females with skewed XCI such that the same X was inactive in all cells. Across the eight skewed samples, 30 genes escaped XCI, 202 genes were subject to XCI and 8 genes variably escaped from XCI (requiring at least two samples with each XCI status to be called variable) (Fig. 2a, Additional file 4: Table S4 for XCI status calls**)**). Two of the genes found to variably escape herein have meta-status calls of variably escaping from XCI, while five were previously designated escaping XCI and one subject to XCI.

The sample-specific XCI status calls determined by allelic expression are more precise; however their use did not substantively alter the histone mark representations found using meta-status calls (Additional file 3: Table S2). The histone marks that were found significant in all three comparisons (CEMT marks vs meta-status, CREST marks vs meta-status, CEMT marks vs CEMT Xi/Xa calls) are H3K4me3 and H3K9me3 (in all but the male XCI comparison), H3K27me3 (between sexes for both XCI statuses), H3K27ac and DNAme (between sexes at genes subject to XCI and between XCI statuses in females), and H3K4me1 and H3K36me3 (only between sexes at genes subject to XCI). An additional benefit of having both histone marks and XCI status on individual samples is the ability to examine how histone marks correlate with XCI status at variably escaping genes.

Genes that variably escape from XCI provide a unique opportunity to study differences between genes escaping vs subject to XCI in the same genomic context. All of the marks available except for H3K4me1 were significantly different (p-value < 0.05) between samples escaping XCI vs those subject to XCI in at least one of the eight variably escaping genes, but never for the majority of genes (Fig. 2b, Additional file 1: Table S5). Consistent with the associations seen for genes subject to or escaping from XCI, when active marks were significantly different, they tended to be higher in samples escaping XCI, while inactive marks were lower in samples escaping XCI (Additional file 1: Table S6). The exception to this is H3K36me3 in gene bodies.

DNAme was the most consistent mark differentiating samples escaping from those subject to XCI, being seen significantly different in four out of the eight variably escaping genes. The samples subject to XCI in *PRKX* had significantly higher DNAme, but were not above the DNAme thresholds for XCI status calls that we established previously [11]. The other three genes with significant DNAme differences showed a clear switch from a DNAme pattern matching genes escaping XCI to a pattern matching genes subject to XCI. *TIMP1*, one of the four genes that was not significant, has low CpG density and high male DNAme so was not expected to differ with XCI status. For the other three genes, the limited informative samples reduced the power to detect differences, although they may have had incorrect XCI status calls or there may be more complicated epigenetic processes involved. Interestingly, the two genes found to be variably escaping by both Xi/Xa expression and meta-status (*MED14* and *TIMP1*) did not show DNAme differences while many of the genes without meta-status calls of variable escape had significant DNAme differences. Thus we have confidence that these other genes (*BCOR, EIF2S3, PRKX* and *SMC1A*) are truly variable across these samples. Three of the variably escaping genes did not show significant differences at any of the examined marks; increasing the sample size might give us the power to see more consistent differences across variably escaping genes as some of these genes only had 2 informative samples per XCI status. Two genes showed significant expression differences between samples that escaped XCI versus those subject to XCI (Additional file 1: Figure S4). In *BCOR*, samples escaping XCI had higher expression across all exons, while in *EIF2S3* some exons were higher in samples subject to XCI while other exons were higher in samples escaping XCI. XCI status and expression per exon may be linked by different TSSs having different XCI status or possibly different tissues having different XCI status and dominant splicing variants. To test whether variable escape may be tissue-specific, XCI status per sample was compared with tissue of origin; only one of the eight genes showed tissue-specificity, *EIF2S3*. However, with only eight samples in three tissue types and being limited by heterozygous polymorphisms, there are likely other variable escape genes that were not identified here as many genes did not have the required number of informative samples.

### Expanding sample-specific XCI status by using DNA methylation

**Fig. 3:**
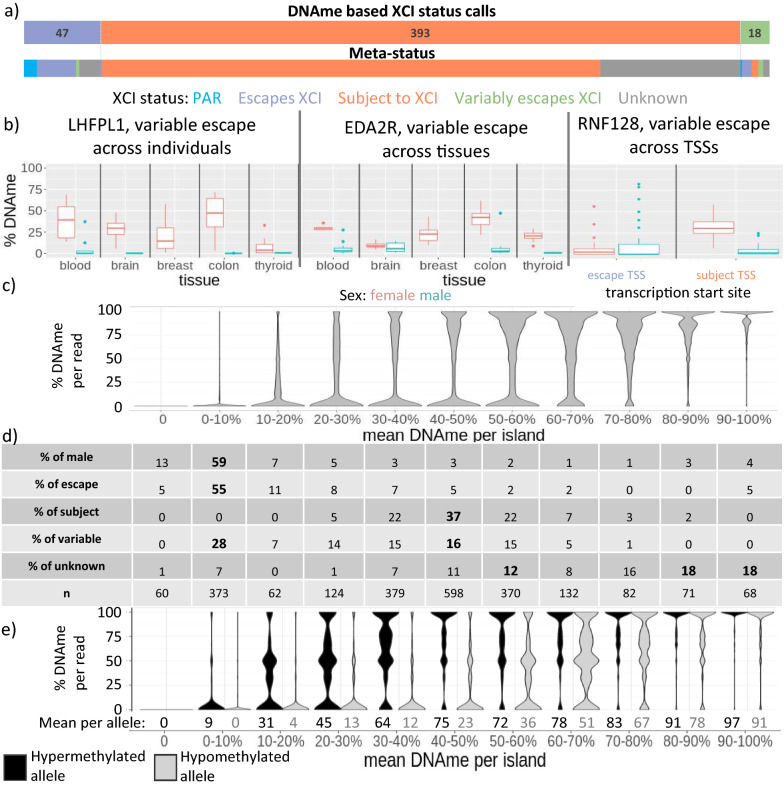
DNAme varies at genes variably escaping from XCI. **a** The number of genes with each XCI status call by DNAme, with their call by meta-status underneath. **b** From left to right: An example of a gene that variably escapes XCI across individuals (and within multiple tissues), a gene that variably escapes from XCI between tissues, and a gene that variably escapes from XCI between TSSs. **c** The percent DNAme per read for genes, binned together by their mean DNAme across the CpG island. Only reads overlapping the CpG island were included here. **d** The distribution of genes with each XCI status across the bins of mean DNAme per island. **e** Allelic DNAme, shown as the percent DNAme per read by allele. The mean DNAme across all reads per allele in each bin is shown underneath

To increase our sample size, we used promoter DNAme levels to determine XCI status across all genes within the larger 45 sample CEMT dataset, regardless of skewed XCI. Only TSSs with high CpG density and low male methylation were considered informative, and within this group we found 47 genes escaping XCI, 393 subject to XCI and 17 variably escaping across samples (Fig. 3a, Additional file 4: Table S4 for XCI status calls). Our DNAme-based calls had strong concordance with meta-status; there were no genes called as escaping XCI here that were previously called as subject to XCI, while only one of the genes called as subject to XCI here was previously called as escaping XCI. We included genes in the variably escaping from XCI category if at least one of their TSSs had 33% or more of its samples escaping XCI and another 33% or more samples subject to XCI. Additionally, one gene had opposing XCI statuses at separate TSSs and 36 had opposite XCI statuses across tissues (examples of genes with these variable escape scenarios are shown in Fig. 3b). An additional 67 genes were found variably escaping in at least one tissue, but were not identified as variably escaping from XCI in the larger dataset. Only *BCOR* was found variably escaping from XCI in the Xi/Xa expression-based calls and also found variably escaping here. In addition 96% of genes escaping and 87% of genes subject to XCI identified by Xi/Xa expression in these samples had concordant status in our DNAme-based calls, with most of the discrepancies between calls being due to genes being called as variably escaping in only one of the datasets.

Comparing epigenetic marks to DNAme-based XCI status calls, all marks (H3K4me3, H3K9me3, H3K27me3 and H3K27ac) except H3K4me1 and H3K36me3 were significantly different between genes with opposite XCI status calls, with increased prominence of H3K9me3 (Additional file 1: Table S7). We again compared epigenetic marks at variably escaping genes to see if they differed between samples in which the gene escaped XCI vs those in which it was subject to XCI. We categorized variable escape genes as those variably escaping across the dataset, across TSSs, across tissues or within specific tissues. For variable escape from XCI between individuals across the dataset, every mark examined was found to be significant (adjusted *p *value < 0.01) in at least one gene; however across all categories of variable escape from XCI, only expression and H3K4me3 were significant in more than 25% of genes in any type of variable escape category (Table 1). The direction of histone mark changes was less consistent than for Xi/Xa expression-based XCI status calls, with the majority of genes still having higher active marks in genes escaping XCI and higher inactive marks in genes subject to XCI, but with many genes showing the opposite results (Additional file 1: Figure S5).

**Table 1:** The percentage of variably escaping (VE) genes found by DNAme that have significant differences in epigenetic marks (BH corrected *p *value < 0.01)

VEtype	n VE transcripts	n VE genes	H3K4me1	H3K4me3	H3K9me3	H3K27ac	H3K27me3	H3K36me3	expression
Across datasets	22	17	6%	17%	18%	17%	17%	16%	0%
Across tissues	70	40	12%	**28%**	7%	16%	14%	21%	**39%**
Between TSSs	2	1	0%	100%	0%	0%	100%	0%	0%
Within blood	74	51	2%	8%	2%	12%	4%	8%	3%
Within brain	10	9	13%	0%	0%	13%	0%	11%	0%
Within breast	29	24	0%	0%	4%	0%	0%	4%	0%
Within colon	8	4	**25%**	**25%**	0%	0%	0%	20%	0%
Within thyroid	45	27	16%	0	7%	10%	0%	24%	0%

Different categories of variable escape are included on the left. The number of variably escaping (VE) transcripts found per category and the number of unique genes are also included. Categories with 25% or more variably escaping genes found significant are bolded, excluding variable escape between TSSs for which only 1 gene was available

We have previously seen that the average DNAme at genes subject to XCI was 38%, less than expected if the Xi were completely methylated, and that some genes subject to XCI had DNAme as low as 15% [11]. It seems likely that the lower female methylation reflects lower Xi methylation, but we further questioned whether lower Xi methylation could predispose genes towards variable escape from XCI. Therefore, we examined the DNAme per WGBS read at CpG islands for the six female samples for which we had WGBS aligned reads, along with one male as a control. The reads for each gene in each sample were subdivided into 10% DNAme bins using the mean DNAme for the gene (Fig. 3c). The genes in the 30–40% DNAme and 40–50% DNAme bins had a surprisingly low number of reads with high DNAme (24% and 35% of reads over 75% DNAme, respectively) so it appears to be that the majority of cells are partially methylated and not that some cells are methylated while others are not (Additional file 1: Table S8). Over 80% of genes with a meta-status of subject to XCI had a mean DNAme between 30 and 60%, while over 60% of genes with a meta-status of escape from XCI and 70% of males across all categories had mean DNAme less than 10% (Fig. 3d). Genes with no known XCI status tended to have high DNAme, with over half of them having 70% DNAme or higher. Intermediate DNAme (reads with 33–66%) is found most frequently in the 20–30% and 70–80% bins. Variably escaping genes were found distributed in the range where genes escaping and subject to XCI were found; however, genes with intermediate 20–30% DNAme had more variably escaping genes than genes with a consistent XCI status.

While the bimodal appearance of the DNAme reads reflects that the Xi and Xa are behaving differently, the intermediate reads could be derived from either. To differentiate DNAme from the Xa and Xi, we examined DNAme per read overlapping heterozygous SNPs within 2 kb of TSSs. In addition to the usual limitations of mapping allelic reads, we had to exclude C <  > T and G <  > A polymorphisms as the bisulfite conversion step in WGBS converts unmethylated C to T and on the opposite strand this appears as a G to A conversion. Separating genes into the same 10% bins of mean DNAme as earlier (Fig. 3e), we see that the intermediately methylated reads tend to be on the hypermethylated allele (the presumed Xi) for bins with less than 40% DNAme and are on the hypomethylated allele for bins with greater than 50% DNAme. We further used this allelic DNAme to call XCI status, using thresholds at 25% and 75% DNAme per allele, with genes having both alleles below 25% being called as escaping from XCI and those having one allele below 25% and one above 75% being called as subject to XCI. These calls for SNPs within CpG islands had good agreement with previous calls with all 28 of the loci called escaping and 50/51 of the loci called subject to XCI being concordant. To explain the prevalence of intermediately methylated reads, we examined the DNAme per CpG across some of these islands where we observed that the DNAme level was not consistent (Additional file 1: Figure S6 for browser tracks across islands, Additional file 1: Figure S7 for DNAme differences between adjacent CpGs). We observe an average difference between adjacent CpG sites of 24% in cancer and 13% in healthy samples, which is likely a major contributor to the intermediately methylated WGBS reads and CpG island DNAme averages we observe for the Xi at these TSSs.

### A combined epigenetic model can predict XCI status across samples

**Fig. 4:**
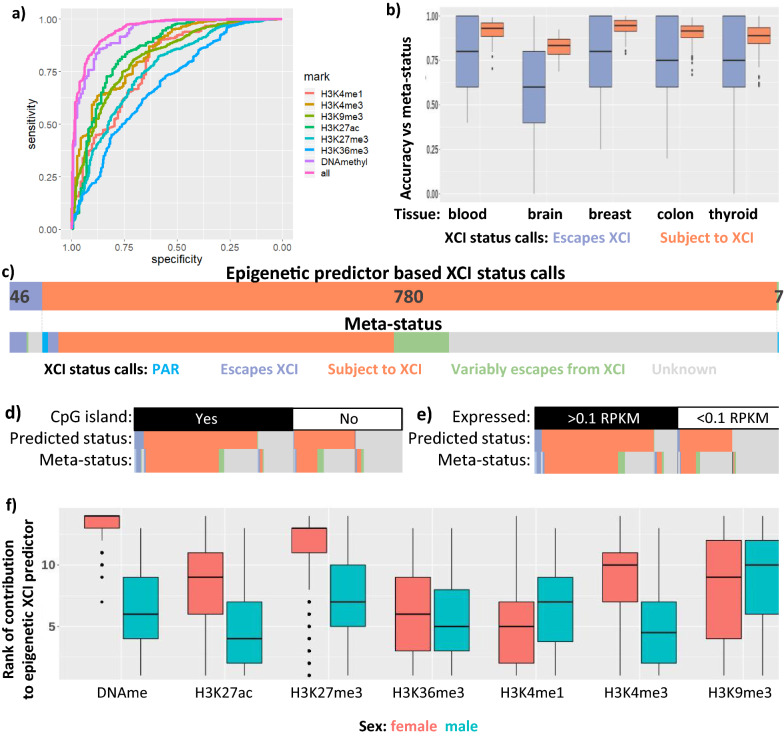
XCI status predictions with an epigenetic model expands the number of genes examinable. **a** ROC curves for each random forest predictor trained using single marks, along with the combined predictor using all of the epigenetic marks. An example sample, CEMT28 is shown. See Additional file 1: Figure S8 for all samples. **b** Accuracy of our epigenetic predictor using DNAme and all six histone marks. Each point is one of the 20 models per sample. This accuracy is tested on genes outside of the training set. **c** The number of genes with each XCI status as predicted by our model, with their call by meta-status underneath. **d**, **e** As (**c**), but further split by the presence of a CpG island (**d**) or by an expression threshold of 0.1 RPKM (**e**). **f** The predictive ability of each mark. Each mark was ranked per model on how important it was to the model, with the most important mark being ranked 14 and the least important being ranked first. We used the marks within each female sample paired with the mean mark in similar male samples for the predictor, so both the female and male marks are featured here

DNAme has been shown repeatedly to be a strong predictor of XCI status, so we wanted to test whether the other epigenetic marks examined could also be used to predict XCI. Using simple thresholds to separate genes that have low values for a mark vs those with high values gave low accuracy and often called genes differently between samples (Additional file 1: Table S9). We moved to a random forest predictor model [34] to predict the XCI status of genes using each individual mark per female sample along with matched male data (Fig. 4a, Additional file 1: Figure S8 for all ROC curves, Additional file 1: Table S10 for accuracies). We trained on 75% of genes escaping XCI, using the remainder to test accuracy, and used twice as many genes subject to XCI for training. Using this predictor, we could predict escape from XCI with accuracies ranging from 42% with H3K9me3 to 69% with H3K4me3 and for genes subject to XCI with accuracies ranging from 85% with genebody H3K36me3 to 99% with H3K27ac. In contrast, a similar model using CpG island DNAme data obtained a much better accuracy of 87% for predicting genes as escaping XCI and 99% for predicting genes as subject to XCI, showing the higher predictive ability of DNAme.

To get XCI status calls from histone mark data with an improved accuracy, we combined data from all of the histone marks and DNAme data from CEMT and trained a new random forest model [35]. This combined epigenetic XCI predictor was trained using XCI meta-status and was able to accurately predict genes escaping vs subject to XCI, with a median accuracy for genes outside the training set of 75% for genes escaping from XCI and 90% for genes subject to XCI (Fig. 4b). We trained the model 20 separate times per sample and were confident in a prediction if 75% + of the models agreed. A separate epigenetic XCI predictor was trained and used within each sample, however the models are capable of being used across samples within the same tissue with reduced accuracy and even across tissues (Additional file 1: Figure S9 for a summary of accuracies). Models in some tissues tended to overcall genes as subject to XCI while others overcalled genes as escaping from XCI, however the number of escape genes called per sample had no correlation with XIST expression (Additional file 1: Figure S10). Across all samples, the model called 46 genes as escaping XCI, 780 genes as subject to XCI and seven genes as variably escaping from XCI (Fig. 4c, Additional file 4: Table S4 for XCI status calls). While none of the genes predicted to escape XCI here have a meta-status of subject to XCI, 11 of the genes predicted to be subject to XCI have a meta-status of escaping XCI and an additional six genes are located in the PAR1 and are expected to escape XCI [2]. Comparing these predictions to our Xi/Xa expression-based XCI status calls, 23 genes escape XCI in both sets while only two were called as escaping XCI by Xi/Xa expression and subject to XCI using this model and three genes had the opposite calls. Of the eight genes found variably escaping by Xi/Xa expression, three of them (CXorf38, PRKX and SMC1A) had their predicted XCI status across samples perfectly match that found by Xi/Xa expression. There are no genes that were given opposite calls across samples by our DNAme-based calls and this model, however some of the genes found to variably escape differed between the two.

This epigenetic XCI predictor can predict XCI status across all genes on the X, without being limited by CpG density and levels of male DNAme that restricted the simple DNAme model; however, transcripts without high promoter CpG density are almost twice as likely to have inconsistent XCI status calls within the same sample while genes that are lowly expressed (median RPKM across samples < 0.1) are over three times more likely to have an inconsistent XCI status call (Fig. 4c, d, Additional file 1: Table S11 for the number of XCI status calls with and without expression and CpG islands). We predicted an XCI status for over 300 genes that did not have previously annotated XCI statuses, however ~ 200 of these had low expression and so may actually be silent on both the active and inactive X making an XCI status call moot. DNAme was the most important input for the models, with H3K27me3 being the next most important (Fig. 4d).

In addition to the seven genes that our epigenetic predictor called as variably escaping XCI across samples, we predicted 48 genes having tissue-specific escape from XCI, and one gene with separate TSSs with opposite XCI status. To investigate which marks are driving this variability in XCI status predictions we compared our epigenetic marks across samples, tissues and TSSs with opposite XCI status predictions (Additional file 1: Table S12). At genes predicted to variably escape across samples we found that very few marks had significant (t-test, adjusted *p *value < 0.01) differences between samples found escaping and those subject to XCI. DNAme was the exception to this with four of seven genes having significant DNAme differences. For the genes found variably escaping across tissues, all of the marks had multiple genes significantly different between tissues subject to XCI vs tissues escaping from XCI, but many of the genes that didn’t variably escape also had significant differences across tissues. Tissue-specific variable escape genes had significant enrichment (Chi-square test, adjusted *p* value < 0.01) for genes with tissue-specific H3K27me3, H3K4me3, DNAme and expression over genes that did not variably escape from XCI. There was only one gene found to variably escape between TSSs so no statistical tests were possible, however there were differences between TSSs for H3K27ac, H3K4me1 and DNAme for the different exons used.

Our initial thresholds to call variable escape across samples were arbitrary, so we varied the percentage of samples with each XCI status required to classify a gene as variably escaping from XCI in order to determine the effects of different variable escape thresholds. At our threshold requiring 33% of samples to have each XCI status in order to be called as variably escaping from XCI, we found 7 of 1155 genes to be variably escaping. Lowering this threshold to 25% found 35 variably escaping genes, at 10% we found 304 genes and at 5% we found 476 genes. This shows that there is no natural threshold at which genes become variable in their expression from the Xi, rather a large proportion of genes will occasionally differ in their XCI status, but few genes are highly variable across samples. As the threshold for calling genes as variably escaping decreased, the percentage of these genes with significant DNAme differences between samples with opposite XCI statuses decreased down to 20% and the percentage of genes with H3K27me3 differences rose to 27% (Additional file 1: Table S13); however, we must also consider that the cancer origin of these samples may contribute to rare epigenetic misregulation.

To validate our conclusions from this model on healthy samples, we trained our overall epigenetic predictor on the CREST dataset. The CREST dataset contains nine samples for which we were able to obtain all of the required epigenetic data for our predictor. We predicted 88 genes escaping from XCI, 802 subject to XCI, 40 variably escaping across samples, ten across tissues and six across TSSs. These calls are similar to those in the CEMT data, with 95% of genes with calls from both datasets agreeing (Additional file 1: Table S14). The genes variably escaping from XCI in the CEMT dataset tended to be escaping XCI in CREST while genes variably escaping in CREST tended to be subject to XCI in the CEMT dataset. The number of genes variably escaping from XCI is increased in CREST, possibly due to how few samples were required for variable escape (three with each XCI status) decreasing stringency. Another possibility is that having random Xi choice doubles the chance of seeing variability in XCI status and the predictor may be sensitive to a change in XCI status in only half of the cells. The number of tissue-specific genes is much reduced in CREST however, likely due to having only two tissues rather than five. Very few of the genes variably escaping across individuals in CREST had significant differences between samples subject to XCI and those escaping from XCI (Additional file 1: Table S15). CREST tissue-specific genes had significant differences in H3K27me3, DNAme and expression between tissues, all three of which were also significant in CEMT samples. CREST had enough genes variably escape across TSSs to see that H3K4me3, H3K27me3 and DNAme were significantly different between TSSs escaping and TSSs subject to XCI in females. Males had significant differences in H3K4me3, H3K27ac, H3K27me3, H3K36me3 and DNAme between TSSs escaping vs subject to XCI in females, which suggests that these TSS also differ significantly on the Xa. These TSSs may be predisposed to have different XCI statuses based on their epigenetic landscape prior to XCI or the Xa differences may be misleading the predictor causing it to predict different XCI statuses. The results between our cancer and healthy samples are similar overall, with results from both datasets finding few genes with significant epigenetic differences between genes variably escaping across individuals, and finding H3K27me3, DNAme and expression differences more commonly different between tissues at genes with a tissue-specific XCI status than at other genes.

### Independent regulation of variable escape across a region

**Fig. 5:**
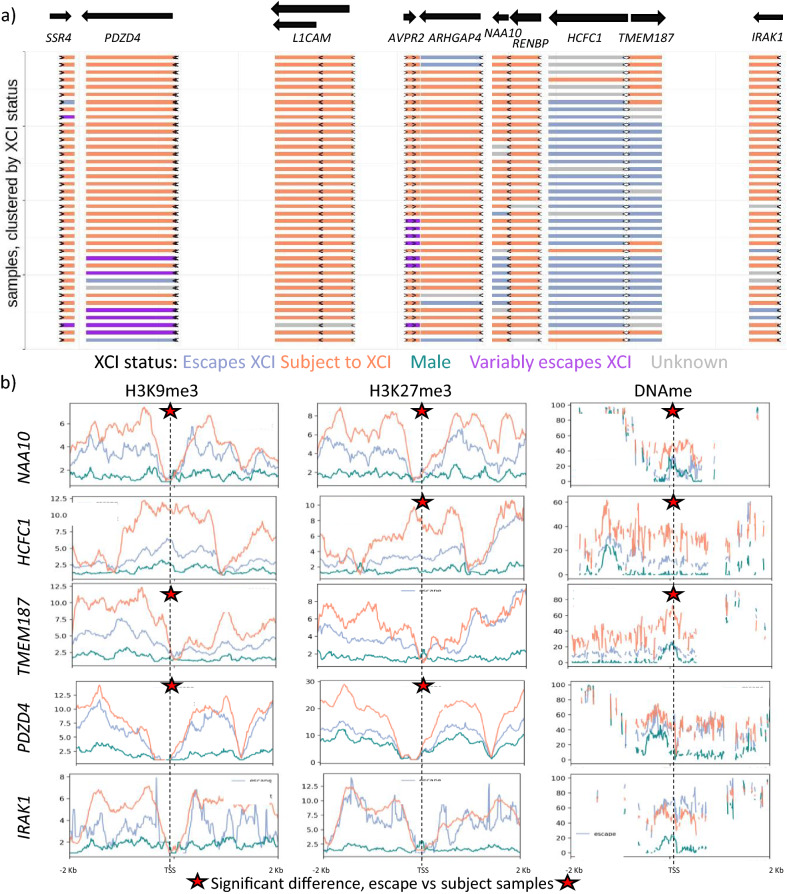
XCI status calls are independent between neighboring variably escaping genes. **a** A map of a variably escaping region, with genes colored by their XCI status as predicted per sample, by our random forest model using all epigenetic marks available. The samples were clustered based on their XCI status calls within the region. Arrows indicate where each TSS is located, and they point in the direction of transcription. Genes which are colored as variably escaping here are variably escaping between transcripts and TSSs within a sample. **b** Metagene plots for the epigenetic marks that were most commonly significantly different between samples subject to XCI vs those escaping from XCI at the above variably escaping genes. Genes were chosen to show every combination of which mark is significant per gene, that we saw in this region. Marks that were significant at a gene are marked with a star

As an application of our epigenetic XCI predictor and to understand the scale at which variably escaping genes are regulated, we examined XCI status calls per sample across a region that is enriched in genes variably escaping from XCI according to their meta-status (Fig. 5a) We found that many of the genes in this region that are annotated as variably escaping from XCI had low levels of variable escape with few samples differing from the most common XCI status. The genes that vary in XCI status across samples change their XCI status independent of the XCI status of neighboring genes, suggesting that regulation of variably escaping genes happens at the single gene level and not at the domain level. Additionally, we saw genes that had multiple TSSs with different XCI statuses and genes that are bidirectional from the same promoter with opposite XCI status showing that the scale of regulation could be narrowed even further. All of the genes in this region that showed variable escape here, except for *IRAK1*, had significant differences for some combination of marks including H3K9me3, H3K27me3 and DNAme between samples escaping vs subject to XCI (*p* value < 0.05, Fig. 5b, Additional file 1: Figure S11 for which marks were significant per TSS). Euchromatic marks were less frequently seen to be significantly different.

### Genetic contribution to variable escape from XCI

**Fig. 6:**
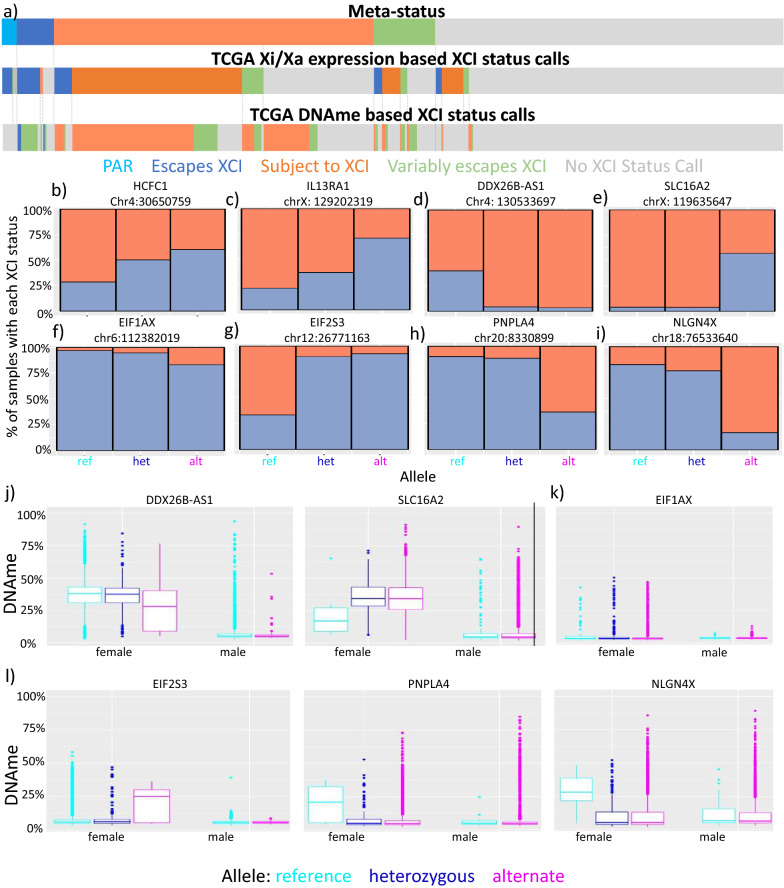
Genetic polymorphisms have an effect on XCI status of variably escaping genes. **a** The number of genes with each XCI status call in the TCGA dataset made using Xi/Xa expression or DNAme, as compared to meta-status calls. Many of the genes escaping XCI by DNAme became variably escaping genes when the threshold for variable escape was lowered to 100 or more samples with each XCI status. **b–i** The percent of samples with each allele that were found with each XCI status at the most significant loci for our association analyses. The chromosomal location below the gene name is for the locus associated with the XCI status of the gene and is the location in hg38. The top row of graphs are the most significant loci associated with Xi/Xa-based XCI status calls from the autosomes (**b**) and X (**c**), along with the top loci associated with DNAme-based XCI status calls from the autosomes (**d**) and X (**e**). The second row is the most significant DNAmeQTL (**f**) and the three DNAmeQTL that are in the range to change XCI status (**g**–**i**). **j**–**l** DNAme values split by sex and allele for top loci by association with DNAme-based XCI status calls (**j**), the most significant DNAmeQTL (**k**), and the DNAmeQTLs in the range to change XCI status (**l**)

To identify any genetic differences at variably escaping genes between samples that escape and those subject to XCI, we obtained existing exome-seq, RNA-seq, Illumina Infinium Human Methylation450 BeadChip array (450 k array) and Affymetrix Genome-Wide Human SNP Array 6.0 (SNP6) data for 5817 samples from cancers where clonality should lead to skewed Xi choice, enabling a comparison of Xi to Xa expression. This data was obtained from The Cancer Genome Atlas (TCGA). We made XCI status calls using both Xi/Xa expression and DNAme to broaden the number of genes and samples available to test for genetic association. We observed 122 genes escaping XCI and 377 genes subject to XCI by Xi/Xa expression. In contrast, DNAme identified 35 genes escaping XCI and 397 genes subject to XCI (Fig. 6a). Of the 25 genes called as escaping from XCI by DNAme that were informative by Xi/Xa expression, all were found escaping by Xi/Xa expression; however, the genes found subject to XCI by DNAme were less concordant, with 222 genes being called as subject to XCI, 19 escaping from XCI and 20 variably escaping from XCI by Xi/Xa expression. Our Xi/Xa calls were limited as we filtered out SNPs with reference bias and samples without skewed XCI; only 1460 samples passed all of our filters. DNAme was limited by the number of genes with CpG islands and with probes on the 450 k array.

Using a threshold requiring 33% of samples to have each XCI status in order to be called as variably escaping from XCI, we found 45 genes variably escaping by Xi/Xa expression but we only found 5 variably escaping genes by DNAme. For our genetic tests, we decided to use a less stringent measure of variable escape for our DNAme calls, as there are so many informative samples here we called any gene with over 100 samples with each XCI status instead of the usual 33% of all samples. This gave us 126 variably escaping genes to test. Of these new variably escaping genes, 26 were previously called as escaping XCI, 59 were called as subject to XCI and 36 did not meet the thresholds for either call previously as too many of the samples were outside of the thresholds to be called as escaping or subject to XCI.

We tested association between XCI status of these variably escaping genes and all SNPs on the SNP6 array and did not find any loci significantly associated with our Xi/Xa expression-based variably escaping genes but with our DNAme-based XCI status calls we found 610 significant combinations of gene and genetic locus across all chromosomes (Additional file 5: Table S16). Only seven of these were X-linked with the closest being 9 Mb away from the affected gene. There were significant loci for 75 of the genes found to variably escape by DNAme, and most of these genes had multiple significant loci, with a maximum of 26 significant loci for *SLC16A2* (Additional file 6: Table S17). Many of the loci were also significantly associated with XCI status for multiple genes, with only 372 unique loci appearing in the 610 significant gene:locus associations. The most genes affected per locus was 18 for chr4:130533697 (Additional file 6: Table S17). However, none of these significant polymorphisms showed 100% correlation with XCI status calls and so they are not the causative or sole-causative polymorphism responsible for the change in XCI status, but may be part of a complex mechanism or be in incomplete linkage disequilibrium with a causative polymorphism (Fig. 6b-i). We examined attributable risk per significant locus and found that the allele with the highest contribution to XCI status had an attributable risk of 28%, but 90% of the loci had attributable risk under 10% (Additional file 5: Table S16). This suggests to us that there are alleles which allow for a change in XCI status and give an increased chance of changing the XCI status, but are not sufficient for the change by themselves.

To test the strength of the effect of SNPs that were significantly associated with XCI status as determined by DNAme, we compared the genotype of samples to their DNAme to find significant DNAme-quantitative trait loci (DNAme-QTL). Testing our 610 significantly associated gene:locus combinations with DNAme-based XCI status calls, we found 38 loci were also significant DNAmeQTLs (Fig. 6j-k, Additional file 7: Table S18). We also tested these DNAmeQTLs in males and all 38 loci were found to only be significant in females. Three of these significant DNAmeQTLs (for the genes *EIF2S3, PNPLA4* and *NLGN4X*) had their median DNAme with one allele in the range to be called as escaping from XCI, with the median DNAme of their other allele in the range to be called as subject to XCI, while the others did not (Fig. 6l). Overall, it appears that there are multiple X-linked and autosomal loci contributing to the variability observed in escape from XCI; however, these are not major contributors and the effect of a single DNAmeQTL is not sufficient for a change in XCI status.

## Discussion

XCI is a classic paradigm for studying epigenetic regulation, yet how some genes are resistant to silencing (or the maintenance of silencing) and escape XCI remains unresolved. Here, we have examined the genetic and epigenetic differences between genes escaping and those subject to XCI. Overall, epigenetic marks were more different between males and females than between genes escaping vs subject to XCI, suggesting an influence of the Xi beyond individual gene regulation. Genes escaping XCI have similar epigenetic marks between the Xi and Xa, except for H3K27me3 which is higher on the Xi. The increased Xi H3K27me3 at genes escaping XCI may be why escape genes can have as low as 10% expression from the Xi compared to the Xa; while the Xa-like status at other marks would allow some expression to continue. The accumulation of H3K27me3 at enhancers on the Xi for both genes subject to and escaping XCI could also contribute to lower expression from the Xi. Overall, genes subject to XCI have higher heterochromatic marks on the Xi and lower euchromatic marks, which supports these genes not being expressed on the Xi. Enhancers were also seen to show strong male:female differences that mostly reflected the presence of the Xi with a lesser contribution from inactivation status. Our results are concordant with previous studies (reviewed in [14]) but broaden both the number of genes and the marks examined.

Across all our epigenetic analyses, DNAme stood out as being the most reflective of a gene’s XCI status. The euchromatic mark H3K4me3 was the histone mark that was most significant for differentiating genes escaping from those subject to XCI, while the heterochromatic mark H3K27me3 had the largest Xi:Xa difference and was the most predictive histone mark for our epigenetic predictor. A previous study, which used a random forest model to predict XCI statuses and silencing timing in mice, found that DNAme often ranked below many of their histone marks, including H3K27ac, H3K4me1 and H3K27me3 [24]. In addition to the possible species differences, their model may not rely on DNAme as much due to the inclusion of genomic and other epigenomic features. A model in humans, which used only genomic repeat elements, achieved an accuracy of 80% for predicting the XCI status of genes [25]. We chose not to incorporate DNA features as we focussed on identifying sample-specific differences in variable escape genes. Our model therefore does not account for the potential interaction of genomic features with the epigenetic marks examined here.

In this study, we used multiple different methods to predict the XCI status of genes and examined how different epigenetic marks changed across genes with differing XCI statuses. We found similar distributions of genes escaping, variably escaping or subject to XCI across our DNAme analyses as our previous Xi/Xa expression analysis [11], while our epigenetic predictor predicted twice as many genes as subject to XCI with similar levels of genes escaping and variably escaping from XCI. A large proportion of the additional genes found subject to XCI by our epigenetic predictor may in fact be silenced on both the Xa and Xi, as 68% of them had a median expression across samples under 0.1 RPKM. The threshold at which to call genes as ‘variable’ in XCI status is arbitrary. We used a threshold requiring 33% of samples to have each XCI status to call variable escape from XCI in our DNAme and epigenetic predictors as used previously [3, 11], with the greater number of samples with each XCI status improving the power of our statistical tests comparing epigenetic marks across samples with opposite XCI statuses. Decreasing the threshold increased the number of genes variably escaping from XCI and the number of epigenetic marks that were significant in at least one gene, but decreased the percentage of genes significant for DNAme that was the only mark ever significant for over 50% of genes in a dataset.

We observed that variable escape from XCI was regulated at the level of single genes, with adjacent genes varying their XCI status independently. In contrast, a study in mice found clusters of genes that variably escape across their three cell lines, with adjacent genes often having the same XCI status across lines [9]. They also found that these clusters colocalize with TADs, with one line having the majority of a TAD escaping XCI and another line having only part of it escaping. An interesting candidate regulator of regional control is *SMCHD1*. In mice with *SMCHD1* knocked-out, regions enriched with variably escaping genes were upregulated, while genes that constitutively escaped from XCI were not affected; however, no impact was seen on variable escape genes in human patients with heterozygous *SMCHD1* mutations [36]. Nonetheless, another study found variants with low expression of *SMCHD1*, *ZSCAN9* and *HBG2/TRIM*6 associated with hypomethylation of X-linked CpG islands, with affected islands enriched near genes that variably escape from XCI [29]. Additionally there are individual genes which are susceptible to reactivation under certain conditions, such as how some genes are reliant on XIST expression and H3K27me3 deacetylation to remain silent, while others continue to be silenced when XIST expression is disrupted [47]. This also supports how our variably escaping genes did not have consistent epigenetic differences between samples which escaped XCI and those which were subject to XCI. Overall, there is evidence for both domain-level and gene-specific regulation of escape. We suggest that for some domains the former predominates, while for other genes the latter predominates. Additionally, the domain featured in Fig. 4 (and other variably escaping domains) is at a threshold where individual genes within the domain can have either XCI status based on local factors.

We thus asked whether variable escape from XCI could be controlled by local sequence variants. Here, we found an association between numerous genetic variants and sample-specific XCI status at variably escaping genes. However, we did not find any local genetic effect, as none of the loci were within 5 Mb of the affected genes and only 10 of 610 significant loci were located on the X. None of the SNPs we identified were completely correlated with a gene’s XCI status, so other factors must be involved. Additionally, all of the significant loci we found were based on DNAme for XCI status calls. These loci could have been affecting just DNAme instead of XCI status, however 38 out of 610 significant loci were female-specific DNAmeQTL while only one loci was a significant DNAmeQTL in males. With more samples with skewed XCI, we may have found loci associated with our Xi/Xa expression-based XCI status calls; the maximum number of informative samples per gene by Xi/Xa expression was 903, while the minimum number of informative samples for our loci associated with DNAme-based XCI status was 2295.

One drawback to this study is that many of our results relied on cancer datasets that may have differences from healthy tissues and epigenetic instability. DNA methyltransferases and histone modifying enzymes are commonly mutated in cancer, and 5–10% of CpG islands that should be unmethylated become methylated (reviewed in [37]). We would expect the changes from epigenetic instability to differ between cancers and cause more genes to variably escape from XCI, however we saw a similar number of genes variably escaping from XCI in the CEMT cancer dataset as in the healthy CREST dataset. Nonetheless, we used the CEMT dataset because it had a standardized set of epigenetic marks across many samples and the clonality of cancer allowed us to examine expression and DNAme allelically. We found that other datasets, did not always have all the marks from the same samples, were lacking females or sex labels or had mislabeled sex.

The use of different methods and sample sizes to call XCI status can result in discordant calls generally due to one approach calling a gene as variably escaping while other studies do not. A previous meta-analysis saw 7% of genes having discordant calls between studies [2]. Many of these discordancies between studies may be due to different samples and tissues used, but here we see differences in XCI status called using different approaches with the same samples. Genes could be falsely called as subject to XCI in the Xi/Xa expression-based analysis if the alternate SNP allele no longer mapped to the same region or if heterozygosity was miscalled. DNAme has been seen misregulated in many cancers [37]. The cancer cells could have mutations mosaic between the parts sampled for different analyses. Our epigenetic predictor did not obtain 100% accuracy on its training data so we expect some of the calls made with it to be false, while the training data could also have false calls further hurting its ability to make accurate XCI status calls.

## Conclusions

In this study, we examined the epigenetic differences (H3K4me1, H3K4me3, H3K9me3, H3K27ac, H3K27me3, H3K36me3, and DNAme) between the Xi and Xa in human cell. Genes subject to XCI had higher heterochromatic marks on the Xi and lower euchromatic marks on the Xi while genes escaping XCI tended to have equal levels of marks on the Xa and Xi, except for H3K27me3 that was consistently high on the Xi. Genes that escape from XCI are not expressed at 100% of the level of the Xa, which may reflect the effect of ongoing heterochromatic mark retention. No mark other than DNAme was very accurate at predicting XCI status; however, combining all of the epigenetic marks together allowed us to robustly call XCI status and also call genes without CpG islands, where DNAme alone is unable to establish a call. Most marks were significantly different between samples escaping vs subject to XCI at variably escaping genes, but which marks were significant was not consistent between genes and no mark was significant across all of the variably escaping genes, likely reflecting that variably escaping genes having multiple ways in which they are regulated. DNAme intermediate to what is expected for genes escaping vs subject to XCI is enriched at variably escaping genes and is mostly due to inconsistent DNAme on the Xi. Neighboring variably escaping genes were seen to regulate their XCI status independently from each other, suggesting local regulatory elements. Additionally, we searched for polymorphisms which may control variable escape from XCI and found non-syntenic loci, some with a strong correlation, but none were completely correlated further suggesting complex regulation. Overall, we see that escape from XCI is influenced by both local regulatory elements as well as trans-acting factors and chromatin modifications that can be independent of each other. Understanding how genes escape from XCI will further our understanding of epigenetics in general and may allow us to control which genes are escaping from XCI and rescue X-linked mutations in females.

## Methods

### Previous XCI status calls

We used XCI meta-status calls from [2] for all comparisons with past XCI statuses and to train our models. Genes that escape and mostly escaped were combined together due to the small size of these categories, with genes in the PAR1 being left out or having their own separate category depending on the analysis. Genes that were mostly subject to XCI were combined with genes subject to XCI for comparisons between studies, but were left out when training models. Genes that were annotated as variably escaping, mostly variably escaping and discordant across studies were combined together as variably escaping genes for comparisons here.

### Histone ChIP-seq analysis

Histone ChIP-seq bigwig files were downloaded from the IHEC data portal [38] and their mean signal quantified with bigWigAverageOverBed [39] for a region 500 bp upstream of TSSs as annotated by Gencode [40]. We normalized the data across samples by multiplying samples to have the same total depth (including all chromosomes). The Xi/Xa ratio was calculated using the following formula (female-male)/male. The metagene plots for Fig. 5 and S2 were generated using Deeptools computeMatrix and plotProfile [41].

### Expression analysis

Xi/Xa expression-based XCI status calls per sample were generated previously [11]. In short, we calculated expression per allele and applied a binomial model [15] to determine whether Xi/Xa expression was above 10% with a 95% confidence interval. The formula used is $\frac{{X_{i} }}{{X_{a} }} \pm \,1.96\sqrt {\frac{{\left( {\frac{{X_{i} }}{{X_{a} }}} \right)\left( {1\frac{{X_{i} }}{{X_{a} }}} \right)}}{{Xi + Xa}},}$ where Xi and Xa are the number of reads per allele and 1.96 is a constant which refers to the alpha required for a 95% confidence interval. In this study, we used a different threshold to identify variably escaping genes, requiring at least two samples with each XCI status. This narrows the number of variably escaping genes and increases the chance that those found would have enough samples to reach significance. The overall expression level of genes was calculated using bigwig files downloaded from the CEEHRC data portal [42] and quantified as RPKM using VisRseq [43].

### DNAme analysis

WGBS bigwig files were downloaded from the IHEC data portal [38] and quantified with bigWigAverageOverBed [39] for a region 500 bp upstream of TSSs as annotated by Gencode [40]. DNAme thresholds established in [11] were used to determine which genes were escaping XCI and which were subject to XCI. These thresholds are: DNAme < 10% escapes XCI, 15% < DNAme < 60% subject to XCI, and DNAme > 60% hypermethylated. A threshold of DNAme < 15% in males was used to filter out TSSs that were methylated on the Xa and therefore not informative for this analysis. To see the differences between adjacent CpGs, we converted bigWig files to bedGraphs and for each island we used R to find the mean absolute value difference between each adjacent CpG.

DNAme per read was calculated by downloading WGBS bam files and using a script to count the number of unmethylated and methylated CG dinucleotides per read within CpG islands within 2 kb of TSSs. For allelic DNAme, we did similar but only examined reads that overlapped heterozygous SNPs identified in our Xi/Xa analysis and had to reconstruct the read from the CIGAR string in the bam file in order to determine the allele of origin. We analyzed allelic DNAme for SNPs within 2 kb of TSSs and noted which were found in CpG islands.

We used bins for every 10% increase in mean DNAme and chose bins for each individual gene per sample separately. All of the reads per bin, across all genes and female samples were used for Fig. 3c. For allelic DNAme we first filtered out polymorphisms where the alleles were CT or GA as bisulfite conversion makes it impossible to differentiate these. We then combined all reads with a C or T allele and all reads with a G or A allele together and filtered out polymorphisms without at least five of each allele type in a sample. The mean DNAme per read per allele type was then calculated and this was used to make XCI status calls per polymorphism in each sample with enough reads. The thresholds were 0.25 and 0.75 for our XCI status calls with polymorphisms with both alleles below 0.25 being called as escaping from XCI and both alleles higher than 0.75 being called as hypermethylated. Polymorphisms with one allele above 0.75 and the other allele below 0.25 were called as subject to XCI. The DNAme per read per polymorphism was binned as above, but instead of using the mean DNAme across all reads, we determined the mean DNAme per allele and used the mean of that; this was done so that we get the mean between the Xi and Xa if there are more reads for one than the other. Additionally, we determined which allele was lower for each polymorphism and graphed the low allele separately from the high allele, per bin.

### Histone-based XCI status predictions

A simple histone predictor was made using genes with known XCI status as published in [2], and defining XCI status for genes within two standard deviations of the mean for each XCI status, similar to a model used in [21]. Because the mean of genes subject to XCI and the mean of genes escaping XCI were often within two standard deviations of each, the average of these two means was often used as a threshold instead.

For our random forest models, we wanted to include both male and female data, and breast did not have any male data so we used the kmeans function in R to cluster all of our samples based on autosomal levels of all seven epigenetic marks used herein. With three clusters we had multiple male and female samples in each cluster. As input for our models, we used individual female data per sample and matched it with the mean values per gene across males in the same cluster.

Random forest models were trained using the R package caret [35] with the trainControl method cv and the train method rf. We trained the model on genes known to escape or be subject to XCI [2]. The training metric was ROC, tunelength was 5 and ntree was 1500. Three genes escaping and subject to XCI were left out of the training set and used to check accuracy of overall calls. We trained twenty models per sample, with each model being trained on a random sample of 75% of the genes escaping XCI and twice as many genes subject to XCI, with each iteration of the model using 75% of the number of input escaping genes. Accuracy per model was tested on the remaining genes with known XCI status. Genes were considered as escaping or subject to XCI if 15 + of 20 models predicted them as escaping or subject to XCI, respectively. Separate categories were made for genes where only 12–14 of the models agreed on the gene’s XCI status, being annotated as leaning subject or leaning escape. Overall calls were made across samples with genes with 66% or more of samples agreeing on a gene’s XCI status being called as subject to or escaping from XCI, genes with at least 33% or more of all samples having each XCI status being called as variably escaping from XCI, and genes that required the leaning categories to reach 66% of samples having a status being annotated with a similar leaning status.

### Statistical comparisons

All statistical comparisons were done in R [44]. The majority were t-tests with a Benjamini–Hochberg (BH) multiple testing correction [45] with results deemed significant if they had an adjusted *p* value < 0.01. The one test with a different threshold was for comparing genes variably escaping XCI as determined by Xi/Xa expression. This test used a 0.05 threshold and had no multi testing correction due to a low sample size, with most genes only having 2 or 3 of each category. If we had a larger sample size, a more stringent test would be preferred. We also used a Chi-square test to determine enrichment of significant histone differences between tissues and TSSs, with *p* value of 0.01.

### TCGA XCI status calls

RNA-seq and exome-seq.bam files were downloaded from the genomic data commons data portal [46] for all female cancer samples in the TCGA datasets. Bcftools mpileup was used to generate bcf files for the exome-seq files, followed by bcftools filter to select for depth 10 + and bcftools call and bcftools view to select heterozygous SNPs with quality over 20.

The RNA-seq data was processed in the following manner: samtools sort and samtools fastq to generate fastq files, followed by realignment by STAR with WASP to limit the effects of reference bias. These new.bam files were then processed similar to the exome-seq data except a depth of 30 + was selected for and there was no filter for heterozygosity.

Using R and our binomial model for Xi/Xa expression used previously in our CEMT expression analysis [11, 15], we then made XCI status calls. Additionally, we filtered out samples that had more than 25% of genes with a meta-status of subject to XCI being called as escaping from XCI, assuming the reason for this is that they did not have skewed XCI.

For the TCGA DNAme-based XCI status calls we downloaded methylation beta-values from the genomic data commons data portal for females and males from the TCGA dataset. Probes were removed if the average male DNAme was over 15% and female samples were removed if their average DNAme was two standard deviations below the female average, as we presume that they were mislabeled males or had lost their Xi. The average DNAme for the filtered probes per gene was then used to make XCI status calls, as has been done previously [11].

### TCGA XCI status association analysis

We tested the association of all SNPs on the SNP6 array vs each variably escaping gene’s XCI status (Chi-square test, significant if BH adjusted *p* value < 0.01). We tested vs all SNPs on the array, and again with just the SNPs on the X. For samples which had multiple SNP array datasets, we used a consensus allele across all of the arrays. We did not include heterozygous samples as we were testing for a cis-effect and had no way of knowing which allele was on the Xi. DNAmeQTLs were examined by using the lm function in R to make a linear model for every combination of SNP and CpG island.

## Supplementary Information

**Additional file1:** **Table S1.** List of samples used. See additional files**. **For CEMT samples, tissue was annotated to combine samples from related areas. Columns D through L refer to the availability of the dataset for each sample. Patient health status and sample disease are the annotations done by CEMT. CREST samples were only used for the epigenetic predictor and only samples with all datasets available were included here. **Table S2.** Comparison of histone marks between sex and XCI status. See additional files**. **The first sheet shows BH adjusted p-values comparing female vs male and escape genes vs those subject to XCI per mark in CEMT with our meta-status and Xi/Xa expression based XCI status calls, along with CREST data with meta-status calls and CEMT data at enhancers with meta-status calls of linked genes. T-tests comparing Xi:Xa ratio per XCI status per dataset are shown on the right. The 2nd sheet shows the median value per mark with each sex and XCI status on the left and on the right shows the Xi/Xa ratio and log2 fold change per mark calculated based off of that median. **Table S3.** The ratio of TSSs with significant differences between males and females for various epigenetic marks using CEMT data.  The denominator was the total number of informative TSSs for which we had data. For most marks this was measured as 500bp upstream of the promoter, but for H3K36me3 we measured the mark across exons. For H3K36me3 we used unique transcripts instead of unique TSSs. Marks significant in over 70% of informative TSSs are in bold. All of the H3K27me3 data from ENCODE was downloaded and used as a replication dataset. Chromosome 7 (chr7) was included as an example autosome. **Table S4.** All XCI status calls made here. See additional files. The first sheet contains a single XCI status call per gene per method. Published calls are from Balaton, et al. 2015. Other sheets contain all calls per sample for each method. Each row is one entry into the model, so Xi/Xa is per gene and the others are for unique transcripts. For DNAme, the samples on the far right in shades of grey are males while the samples on the left in color are females. For the epigenetic predictor, separate low confidence categories were made for when transcripts have only 12-14 of the 20 models per sample predicted a certain XCI status. Start and stop locations are from hg38. **Table S5.** Significance of differences in epigenetic marks between samples with opposite XCI statuses at genes found variably escaping XCI by Xi/Xa expression. We also tested whether expression differed between samples with opposite XCI status. Presented here are the p-values of t-tests. Those with p-values less than 0.05 are in bold. nE and nS are the number of samples escaping or subject to XCI for each gene. **Table S6.** The differences in epigenetic marks between samples with opposite XCI statuses at genes found variably escaping XCI by Xi/Xa expression. The mean value for samples subject to XCI was subtracted from the mean value for samples escaping XCI. Those found significant in Table S5 are bolded. Genes with multiple transcripts are included multiple times, even if they share a TSS. **Table S7.** Adjusted p-values comparing marks in females between genes found subject to XCI vs escaping XCI by DNAme. Those in bold are significant (adjusted p-value<0.01). **Table S8.** Distribution summary for DNAme per read. The number is what proportion of reads in each bin were below 25%, between 33 and 66% or over 75% DNAme. **Table S9.** The accuracy of simple models predicting XCI status from a single histone mark. These accuracies are low because the models overpredicted variable escape from XCI as there is large overlap between the two XCI statuses. **Table S10.** The accuracy of random forest models predicting XCI status from a single histone mark. This is the combined accuracy using the consensus of 20 models trained with each mark. **Table S11.** XCI status calls made using a random forest epigenetic predictor, split by presence or absence of a CpG island and expression. The threshold used to split low from high expression is a median of 0.1 RPKM across samples. Inconsistent predictions had over a third of samples with fewer than 15 of the 20 models trained agree on an XCI status. **Table S12.** The percent of genes found variably escaping by our epigenetic predictor with significant differences in various epigenetic marks. Genes were counted as significant if BH corrected p-values were less than 0.01 when using t tests to compare samples predicted as subject to XCI to samples predicted as escaping from XCI. The total number of genes row shows the total number of genes in each category. The variable escape across tissues and TSSs categories have 2 columns each, the left column being the percent of variably escaping genes with significant differences between tissues/TSSs and the right column being the percent of all genes on the X with differences between tissues/TSSs. Highlighted in blue are marks that were significantly more likely to have significant differences between tissues/TSSs at genes predicted to variably escape than in all X linked genes (Chi-square adjusted p-value<0.01). **Table S13.** The percent of genes found variably escaping by our epigenetic predictor with significant differences in various epigenetic marks across various variable escape thresholds. Variable escape threshold is the number of samples with each XCI status (escaping from XCI and subject to XCI) that were required in order to call a gene as variably escaping from XCI across samples. Genes were counted as significant if BH corrected p-values were less than 0.01 when comparing samples predicted as subject to XCI to samples predicted as escaping from XCI. **Table S14.** Comparing XCI status calls made by an epigenetic predictor in the CEMT dataset vs a similar model in the CREST dataset. **Table S15.** The percent of genes found variably escaping by our epigenetic predictor in the CREST dataset with significant differences in various epigenetic marks. Genes were counted as significant if BH corrected p-values were less than 0.01 when using t tests to compare samples predicted as subject to XCI to samples predicted as escaping from XCI. The total number of genes row shows the total number of genes in each category. The variable escape across tissues and TSSs categories have 2 columns each, the left column being the percent of variably escaping genes with significant differences between tissues/TSSs and the right column being the percent of all genes on the X with differences between tissues/TSSs. Highlighted in blue are marks that were significantly more likely to have significant differences between tissues/TSSs at genes predicted to variably escape than in all X linked genes (Chi-square adjusted p-value<0.01). **Table S16.** Top 100 results from an analysis associating XCI status with genotype. See additional files. There are separate sheets for association with Xi/Xa and 450k based XCI status calls, and for comparing to all chromosomes, and just chromosome X. The adjusted p-value is calculated using the Benjamini-Hochberg method. For the sheet associating DNAme based XCI status calls with loci on all chromosomes, we included all 610 significant loci instead of the top 100. I have also included the amount of samples with each XCI status (E for escapes XCI, S for subject to XCI) and each genotype (ref for reference allele, het for  heterozygous, alt for alternate allele) (columns E-J). Columns M-N are the ratio of reference to alternate alleles at samples escaping or subject to XCI, with O being the ratio of these two columns and P being the reciprocal of O if it is less than 1, to make comparison easier. This enrichment column (col P) shows enrichment of reference allele at samples with one XCI status over the other. For the DNAme allChr sheet we have also included a column showing the attributable risk per allele. **Table S17.** The number of loci associated with each gene and genes associated with each locus. See additional files. These are for the association between DNAme based XCI status and genetic polymorphisms. **Table S18.** DNAmeQTL analysis for the loci significantly associated with DNAme-based XCI status calls. See additional files. These loci were independently tested as DNAmeQTLs in females and males, with some columns color coded based on sex (pink female, light blue male). There are also columns with the median and mean DNAme value at the gene’s island for samples with the reference or alternate allele at that loci; these columns are color coded based on whether the allele is in the range to escape from XCI (DNAme<0.01, blue) or in the range to be subject to XCI (DNAme>0.15, orange). There are mean and median columns for both males and females, but only the female columns are color coded based on XCI status. There are boxes around the genes with female median values with one allele in the range to escape XCI and the other allele in the range to be subject to XCI. **Figure S1.** log2(Xi/Xa) for epigenetic marks in CREST. See Table S2 for which comparisons are significant. **Figure S2.** Meta-gene plots of histone marks within 50kb of genes, separated by XCI status. The plots were generated using deeptools computeMatrix and plotProfile on bigwig files that were the mean across samples. Solid lines show the mean values per gene, after having averaged each gene across samples. Lighter shaded regions show the standard deviation of each mark. **Figure S3.** log2(Xi/Xa) for epigenetic marks in CEMT at enhancers mapping to genes that escape from or are subject to XCI. Enhancers are split by whether they are located within a gene (genic) or not (intergenic). **Figure S4.** Expression across exons for genes with significantly different expression in samples with opposite XCI statuses. XCI status per sample was determined here using Xi/Xa expression. **Figure S5.** Differences in epigenetic marks between samples found escaping vs subject to XCI at variably escaping genes called using DNAme. For most of these marks, the region 500bp upstream of the promoter is used, except for H3K36me3 which uses the gene body. The median value per gene in samples found subject to XCI was subtracted from the median value per gene in samples which escaped from XCI. This is done here for all genes found variably escaping across individuals by DNAme. **Figure S6.** IGV view of DNAme bigwig tracks at two variably escaping genes. a) A view of the CpG island at CITED1. b) a view of the CpG island at NAA10. A broad representation of samples was sought, some hypomethylated, some hypermethylated and some inconsistent across the CpG island. Broad hypermethylation in males at these genes was rare but is included here as an example of an extreme. **Figure S7.** Average DNAme difference between adjacent CpGs per CpG island. Each point is the average DNAme difference between adjacent CpGs for an individual island, averaged again across samples. Islands are colored by the meta-status of the closest TSS within 2kb. Chr7 was chosen as an autosomal control to show whether the differences are X specific. Males and females from CEMT were used to check for sex specificity and females from CREST were included to check for cancer specificity. **Figure S8.** ROC for predictive models trained with each epigenetic mark. On display is one random forest model trained per sample with one epigenetic mark as its input, along with the median value of the mark in similar males. Samples are colored by tissue. The all category is for a predictor using all 6 histone marks and DNAme. Black diagonal lines were added to ease comparison between figures.**Figure S9. **Accuracy when models trained in one sample are tested on other models.** Figure S10.** Comparing XIST expression to the number of escape genes predicted per sample. Predictions were made using a random forest model with all histone marks and DNAme. **Figure S11.** Which marks were significantly different between samples predicted as escaping vs subject to XCI in a variably escaping region. Transcript ID is the order that the transcripts are located along the chromosome. There are multiple transcripts per gene but they may be sharing the same TSS and have the same data for all marks but H3K36me3. Vertical lines are drawn denoting which transcripts belong with each gene.

**Additional file2:** **Table S1.** List of samples used. See additional files**. **For CEMT samples, tissue was annotated to combine samples from related areas. Columns D through L refer to the availability of the dataset for each sample. Patient health status and sample disease are the annotations done by CEMT. CREST samples were only used for the epigenetic predictor and only samples with all datasets available were included here.

**Additional file3:** **Table S2.** Comparison of histone marks between sex and XCI status. See additional files**. **The first sheet shows BH adjusted p-values comparing female vs male and escape genes vs those subject to XCI per mark in CEMT with our meta-status and Xi/Xa expression based XCI status calls, along with CREST data with meta-status calls and CEMT data at enhancers with meta-status calls of linked genes. T-tests comparing Xi:Xa ratio per XCI status per dataset are shown on the right. The 2nd sheet shows the median value per mark with each sex and XCI status on the left and on the right shows the Xi/Xa ratio and log2 fold change per mark calculated based off of that median. 

**Additional file4:** **Table S4.** All XCI status calls made here. See additional files. The first sheet contains a single XCI status call per gene per method. Published calls are from Balaton, et al. 2015. Other sheets contain all calls per sample for each method. Each row is one entry into the model, so Xi/Xa is per gene and the others are for unique transcripts. For DNAme, the samples on the far right in shades of grey are males while the samples on the left in color are females. For the epigenetic predictor, separate low confidence categories were made for when transcripts have only 12-14 of the 20 models per sample predicted a certain XCI status. Start and stop locations are from hg38.

**Additional file5:** **Table S16.** Top 100 results from an analysis associating XCI status with genotype. See additional files. There are separate sheets for association with Xi/Xa and 450k based XCI status calls, and for comparing to all chromosomes, and just chromosome X. The adjusted p-value is calculated using the Benjamini-Hochberg method. For the sheet associating DNAme based XCI status calls with loci on all chromosomes, we included all 610 significant loci instead of the top 100. I have also included the amount of samples with each XCI status (E for escapes XCI, S for subject to XCI) and each genotype (ref for reference allele, het for  heterozygous, alt for alternate allele) (columns E-J). Columns M-N are the ratio of reference to alternate alleles at samples escaping or subject to XCI, with O being the ratio of these two columns and P being the reciprocal of O if it is less than 1, to make comparison easier. This enrichment column (col P) shows enrichment of reference allele at samples with one XCI status over the other. For the DNAme allChr sheet we have also included a column showing the attributable risk per allele.

**Additional file6:** **Table S17.** The number of loci associated with each gene and genes associated with each locus. See additional files. These are for the association between DNAme based XCI status and genetic polymorphisms.

**Additional file7:** **Table S18.** DNAmeQTL analysis for the loci significantly associated with DNAme-based XCI status calls. See additional files. These loci were independently tested as DNAmeQTLs in females and males, with some columns color coded based on sex (pink female, light blue male). There are also columns with the median and mean DNAme value at the gene’s island for samples with the reference or alternate allele at that loci; these columns are color coded based on whether the allele is in the range to escape from XCI (DNAme<0.01, blue) or in the range to be subject to XCI (DNAme>0.15, orange). There are mean and median columns for both males and females, but only the female columns are color coded based on XCI status. There are boxes around the genes with female median values with one allele in the range to escape XCI and the other allele in the range to be subject to XCI.

## Data Availability

See references for data sources. All are publicly available.

## Abbreviations

450k array: Illumina Infinium Human Methylation450 BeadChip array
BH: Benjamini–Hochberg
CEMT: Center for Epigenome Mapping Technologies
ChIP-seq: Chromatin immunoprecipitation sequencing
CREST: Core Research for Evolutional Science and Technology
DNAme: DNA methylation
DNAmeQTL: DNA methylation quantitative trait loci
IHEC: International Human Epigenome Consortium
meta-status: XCI status calls from Balaton et al., 2015
PAR: Pseudo-autosomal region
RNA-seq: RNA sequencing
SNP6: Affymetrix Genome-Wide Human SNP Array 6.0
TAD: Topologically associating domain
TCGA: The Cancer genome Atlas
TSS: Transcription start site
WGBS: Whole genome bisulfite sequencing
X: X chromosome
Xa: Active X
XCI: X-chromosome inactivation
Xi: Inactive X
