# Supplementary material for: Contribution of genetic and epigenetic changes to escape from X-chromosome inactivation
Source: Epigenetics Chromatin. 2021 Jun 29;14:30. doi: 10.1186/s13072-021-00404-9 (PMC8244145; doi:10.1186/s13072-021-00404-9)
Supplement: Supplementary file 1 — Additional file1: Table S1. List of samples used. See additional files. For CEMT samples, tissue was annotated to combine samples from related areas. Columns D through L refer to the availability of the dataset for each sample. Patient health status and sample disease are the annotations done by CEMT. CREST samples were only used for the epigenetic predictor and only samples with all datasets available were included here. Table S2. Comparison of histone marks between sex and XCI status. See additional files. The first sheet shows BH adjusted p-values comparing female vs male and escape genes vs those subject to XCI per mark in CEMT with our meta-status and Xi/Xa expression based XCI status calls, along with CREST data with meta-status calls and CEMT data at enhancers with meta-status calls of linked genes. T-tests comparing Xi:Xa ratio per XCI status per dataset are shown on the right. The 2nd sheet shows the median value per mark with each sex and XCI status on the left and on the right shows the Xi/Xa ratio and log2 fold change per mark calculated based off of that median. Table S3. The ratio of TSSs with significant differences between males and females for various epigenetic marks using CEMT data. The denominator was the total number of informative TSSs for which we had data. For most marks this was measured as 500bp upstream of the promoter, but for H3K36me3 we measured the mark across exons. For H3K36me3 we used unique transcripts instead of unique TSSs. Marks significant in over 70% of informative TSSs are in bold. All of the H3K27me3 data from ENCODE was downloaded and used as a replication dataset. Chromosome 7 (chr7) was included as an example autosome. Table S4. All XCI status calls made here. See additional files. The first sheet contains a single XCI status call per gene per method. Published calls are from Balaton, et al. 2015. Other sheets contain all calls per sample for each method. Each row is one entry into the model, so Xi/Xa is per gene and the [file 13072_2021_404_MOESM1_ESM.pdf]

Supplemental Tables:

Table S1: List of samples used. See additional files.For CEMT samples, tissue was manually annotated to combine samples from related areas. Columns D through L are true if the dataset was available for the sample. Patient health status and sample disease are the annotations done by CEMT. CREST samples were only used for the epigenetic predictor and only samples with all datasets available were included here.

Table S2: Comparison of histone marks between sex and XCI status. See additional files. The first sheet shows BH adjusted p-values comparing female vs male and escape genes vs those subject to XCI per mark in CEMT with our meta-status and Xi/Xa expression based XCI status calls, along with CREST data with meta-status calls and CEMT data at enhancers with meta-status calls of linked genes. T-tests comparing Xi:Xa ratio per XCI status per dataset are shown on the right. The 2nd sheet shows the median value per mark with each sex and XCI status on the left and on the right shows the Xi/Xa ratio and log2 fold change per mark calculated based off of that median.

	H3K4me1	H3K4me3	H3K9me3	H3K27ac	H3K27me3	ENCODE H3K27me3	H3K36me3	DNAme
**escapes from XCI**	0.45	0.56	0.47	0.08	**0.97**	**0.72**	0.46	0.26
**no previous call**	0.28	0.15	0.66	0.01	**0.87**	0.49	0.30	0.29
**PAR**	0	0.00	0.28	0.00	0.04	0.11	0.17	0.04
**subject to XCI**	0.10	0.07	**0.88**	0.01	**0.90**	**0.83**	0.32	**0.80**
**variably escapes from XCI**	0.15	0.09	**0.80**	0.01	**0.93**	**0.93**	0.30	0.54
**chr7**	0.15	0.03	0.05	0.08	0.10	0.17	0.07	0.30

Table S3: The ratio of TSSs with significant differences between males and females for various epigenetic marks using CEMT data. The denominator was the total number of informative TSSs for which we had data. For most marks this was measured as 500bp upstream of the promoter, but for H3K36me3 we measured the mark across exons. For H3K36me3 we used unique transcripts instead of unique TSSs. Marks significant in over 70% of informative TSSs are in bold. All of the H3K27me3 data from ENCODE was downloaded and used as a replication dataset. Chromosome 7 (chr7) was included as an example autosome.

Table S4: All XCI status calls made here. See additional files. The first sheet contains a single XCI status call per gene per method. Published calls are from Balaton, *et al*. 2015. Other sheets contain all calls per sample for each method. Each row is one entry into the model, so Xi/Xa is per gene and the others are for unique transcripts. For DNAme, the samples on the far right in shades of grey are males while the samples on the left in color are females. For the epigenetic predictor, separate low confidence categories were made for when transcripts have only 12-14 of the 20 models per sample predicted a certain XCI status. Start and stop locations are from hg38.

gene	H3K4me1	H3K4me3	H3K9me3	H3K27ac	H3K27me3	H3K36me3	DNAme	expression	nE	nS
BCOR	0.076	0.42	**0.019**	0.43	0.065	0.56	**0.011**	**0.0097**	2	5
CXorf38	0.071	0.26	0.82	0.52	0.18	0.24	0.10	0.18	3	2
EIF2S3	0.054	0.097	0.80	0.12	0.33	0.54	**0.040**	0.070	4	2
MED14	0.66	0.98	0.32	0.39	0.28	0.45	0.42	0.86	2	3
PNPLA4	0.84	**0.029**	**0.0076**	0.069	0.070	0.42	0.15	0.74	4	2
PRKX	0.73	0.070	**0.029**	**0.047**	0.27	**0.021**	**0.048**	0.053	5	3
SMC1A	0.11	**0.043**	0.24	0.61	**0.036**	**0.048**	**0.046**	0.15	6	2
TIMP1	0.59	0.40	0.55	0.054	0.78	0.18	0.21	0.87	3	4

Table S5: Significance of differences in epigenetic marks between samples with opposite XCI statuses at genes found variably escaping XCI by Xi/Xa expression. We also tested whether expression differed between samples with opposite XCI status. Presented here are the p-values of t-tests. Those with p-values less than 0.05 are in bold. nE and nS are the number of samples escaping or subject to XCI for each gene.

gene	H3K4me1	H3K4me3	H3K9me3	H3K27ac	H3K27me3	H3K36me3	DNAme	expression
PRKX	2.207482	10.81617	**-2.32661**	**4.171272**	-2.94785	**1.586497**	**-4.70569**	0.460988
PNPLA4	1.159099	**10.4815**	-14.1268	8.604572	-3.90181	-1.2279	-17.5906	0.887235
PNPLA4	1.088554	6.106866	**-7.22367**	3.873968	-0.5687	-1.2279	-13.0625	0.79953
PNPLA4	1.088554	6.106866	**-7.22367**	3.873968	-0.5687	-0.50595	-13.0625	1.070425
EIF2S3	4.328741	12.59848	0.666695	-7.88552	-2.50986	0.42919	**-30.1127**	56.54015
BCOR	2.831612	-1.35245	-0.25747	1.548647	1.395386	-0.26325	15.31005	**32.60517**
BCOR	5.870941	1.363537	**-0.54052**	1.472802	-5.61021	-0.26325	**-22.549**	**33.10998**
BCOR	5.870941	1.363537	**-0.54052**	1.472802	-5.61021	0.016699	**-22.549**	**32.64655**
CXorf38	13.65249	3.909036	-1.12366	1.58118	-3.6874	-0.11738	-54.6914	10.16366
CXorf38	16.67418	2.714651	-0.85511	1.846735	-4.25266	-0.35507	-36.7491	10.28184
MED14	1.039564	3.233193	-1.08359	0.868199	-3.15195	-0.18656	-0.58621	0.365396
TIMP1	3.746742	-0.83792	0.437359	-0.83214	-1.3243	-0.34116	14.47131	0.016658
SMC1A	-8.21266	**8.614903**	-9.708	1.579612	**-5.34306**	**-0.22771**	**-43.8589**	-0.06352
SMC1A	-8.21266	**8.614903**	-9.708	1.579612	**-5.34306**	-0.1797	**-43.8589**	-0.08209

Table S6: The differences in epigenetic marks between samples with opposite XCI statuses at genes found variably escaping XCI by Xi/Xa expression. The mean value for samples subject to XCI was subtracted from the mean value for samples escaping XCI. Those found significant in Table S5 are bolded. Genes with multiple transcripts are included multiple times, even if they share a TSS.

**H3K4me1**	0.093906
**H3K4me3**	**3.34E-11**
**H3K9me3**	**7.66E-68**
**H3K27ac**	**8.11E-09**
**H3K27me3**	**1.03E-29**
**H3K36me3**	0.067493

Table S7: adjusted p-values comparing marks in females between genes found subject to XCI vs escaping XCI by DNAme. Those found significant are in bold (adjusted p-value<0.01).

bin	<25%	33-66%	>75%
**0**	0.99	0.00	0.00
**0-10%**	0.97	0.01	0.00
**10-20%**	0.73	0.14	0.04
**20-30%**	0.59	0.17	0.13
**30-40%**	0.52	0.13	0.24
**40-50%**	0.45	0.09	0.36
**50-60%**	0.34	0.11	0.44
**60-70%**	0.21	0.15	0.50
**70-80%**	0.08	0.17	0.60
**80-90%**	0.03	0.09	0.75
**90-100%**	0.01	0.02	0.91

Table S8: Distribution summary for DNAme per read. The number is what percent of reads in each bin were below 25%, between 33 and 66% or over 75% DNAme.

Histone Mark	Accuracy for genes escaping XCI	Accuracy for genes subject to XCI
**H3K4me1**	0.27	0.45
**H3K4me3**	0.36	0.55
**H3K9me3**	0.11	0.16
**H3K27ac**	0.33	0.64
**H3K27me3**	0.51	0.21
**H3K36me3**	0.09	0.03

Table S9: The accuracy of simple models predicting XCI status from a single histone mark. These accuracies are low because the models overpredicted variable escape from XCI as there is large overlap between the two XCI statuses.

Histone Mark	Accuracy for genes escaping XCI	Accuracy for genes subject to XCI
**H3K4me1**	0.67	0.88
**H3K4me3**	0.69	0.97
**H3K9me3**	0.42	0.97
**H3K27ac**	0.63	0.99
**H3K27me3**	0.60	0.96
**H3K36me3**	0.58	0.85
**DNAme**	0.87	0.99

Table S10: The accuracy of random forest models predicting XCI status from a single histone mark. This is the combined accuracy using the consensus of 20 models trained with each mark.

	No CpG island	CpG island	low expression	high expression
**escapes XCI**	8	95	26	77
**subject to XCI**	600	1116	549	1167
**variably escapes XCI**	2	7	1	8
**inconsistent prediction**	462	346	569	239

Table S11: XCI status calls made using a random forest epigenetic predictor with all of the epigenetic marks, split by presence or absence of a CpG island and expression. The threshold used to split low from high expression is a median of 0.1 RPKM across samples. Inconsistent predictions had over a third of samples with fewer than 15 of the 20 models trained agree on an XCI status.

	Variable escape across individuals	Variable escape across tissues		Variable escape across TSSs	
**Total number of genes**	**9**	**65**	**2636**	**1**	**461**
**H3K27me3 TSS**	0	**51%**	27%	0	12%
**H3K27ac TSS**	0	31%	37%	100%	17%
**H3K9me3 TSS**	0	32%	19%	0	30%
**H3K4me3 TSS**	0	**40%**	20%	0	22%
**H3K4me1 TSS**	0	62%	53%	100%	19%
**H3K36me3 genebody**	11%	29%	31%	0	38%
**DNAme TSS**	67%	**58%**	28%	100%	19%
**expression**	0	**38%**	1%		

Table S12: The percent of genes found variably escaping by our epigenetic predictor with significant differences in various epigenetic marks. Genes were counted as significant if BH corrected p-values were less than 0.01 when using t tests to compare samples predicted as subject to XCI to samples predicted as escaping from XCI. The total number of genes row shows the total number of genes in each category. The variable escape across tissues and TSSs categories have 2 columns each, the left column being the percent of variably escaping genes with significant differences between tissues/TSSs and the right column being the percent of all genes on the X chromosome with differences between tissues/TSSs. Highlighted in blue are marks which were significantly more likely to have significant differences between tissues/TSSs at genes predicted to variably escape than in all X linked genes (Chi-square adjusted p-value<0.01).

**variable escape threshold**	**33%**	**25%**	**10%**	**5%**
**Number of genes variably escaping across samples**	9	41	431	740
**H3K27me3 TSS**	0%	4.90%	23%	27%
**H3K27ac TSS**	0%	2%	3.50%	7.80%
**H3K9me3 TSS**	0%	0%	11%	15%
**H3K4me3 TSS**	0%	0%	2.80%	4.20%
**H3K4me1 TSS**	0%	4.90%	7.70%	10%
**H3K36me3 genebody**	11%	7%	3.50%	6.20%
**DNAme TSS**	67%	22%	17%	20%
**expression**	0%	0%	0.90%	0.70%

Table S13: The percent of genes found variably escaping by our epigenetic predictor with significant differences in various epigenetic marks across various variable escape thresholds. Variable escape threshold is the number of samples with each XCI status (escaping from XCI and subject to XCI) that were required in order to call a gene as variably escaping from XCI across samples. Genes were counted as significant if BH corrected p-values were less than 0.01 when comparing samples predicted as subject to XCI to samples predicted as escaping from XCI.

		CEMT calls per gene		
		Escapes XCI	Subject to XCI	Variably escapes XCI
CREST calls per gene	Escapes XCI	64	37	7
	Subject to XCI	3	1492	1
	Variably escapes XCI	1	32	0

Table S14: Comparing XCI status calls made by an epigenetic predictor in the CEMT dataset vs a similar model in the CREST dataset.

	VE across individuals	VE across tissues		VE across TSSs	
**total**	**53**	**13**	**2313**	**6**	**1155**
**H3K4me1.female**	0	0	0.076%	0	0.087%
**H3K4me1.male**	NA	NA	NA	17%	4.2%
**H3K4me3.female**	0	0	0	**50%**	1%
**H3K4me3.male**	NA	NA	NA	**67%**	6%
**H3K9me3.female**	0	0	0	0	0
**H3K9me3.male**	NA	NA	NA	33%	5%
**H3K27ac.female**	0	0	0.08%	17%	1%
**H3K27ac.male**	NA	NA	NA	**50%**	5%
**H3K27me3.female**	0	**15%**	**0.11%**	**17%**	9%
**H3K27me3.male**	NA	NA	NA	**33%**	2%
**H3K36me3.female**	0	0	0	0	2%
**H3K36me3.male**	NA	NA	NA	**50%**	3%
**DNAme.female**	2%	**92%**	**2.31%**	**100%**	6%
**DNAme.male**	NA	NA	NA	**100%**	1%
**RNAseq.female**	4%	**46%**	**1.33%**	0	3%
**RNAseq.male**	NA	NA	NA	17%	3%

Table S15: The percent of genes found variably escaping by our epigenetic predictor in the CREST dataset with significant differences in various epigenetic marks. Genes were counted as significant if BH corrected p-values were less than 0.01 when using t tests to compare samples predicted as subject to XCI to samples predicted as escaping from XCI. The total number of genes row shows the total number of genes in each category. The variable escape across tissues and TSSs categories have 2 columns each, the left column being the percent of variably escaping genes with significant differences between tissues/TSSs and the right column being the percent of all genes on the X chromosome with differences between tissues/TSSs. Highlighted in blue are marks which were significantly more likely to have significant differences between tissues/TSSs at genes predicted to variably escape than in all X linked genes (Chi-square adjusted p-value<0.01).

Table S16: Top 100 results from an analysis associating XCI status with genotype. See additional files. There are separate sheets for association with Xi/Xa and 450k based XCI status calls, and for comparing to all chromosomes, and just chromosome X. The adjusted p-value is calculated using the Benjamini-Hochberg method. For the sheet associating DNAme based XCI status calls with loci on all chromosomes, we included all 610 significant loci instead of the top 100. I have also included the amount of samples with each XCI status (E for escapes XCI, S for subject to XCI) and each genotype (ref for reference allele, het for heterozygous, alt for alternate allele) (columns E-J). Columns M-N are the ratio of reference to alternate alleles at samples escaping or subject to XCI, with O being the ratio of these two columns and P being the reciprocal of O if it is less than 1, to make comparison easier. This enrichment column (col P) shows enrichment of reference allele at samples with one XCI status over the other. For the DNAme allChr sheet we have also included a column showing the attributable risk per allele.

Table S17: The number of loci associated with each gene and genes associated with each locus. See additional files. These are for the association between DNAme based XCI status and genetic polymorphisms.

Table S18: DNAmeQTL analysis for the loci significantly associated with DNAme-based XCI status calls. See additional files. These loci were independently tested as DNAmeQTLs in females and males, with some columns color coded based on sex (pink female, light blue male). There are also columns with the median and mean DNAme value at the gene's island for samples with the reference or alternate allele at that loci; these columns are color coded based on whether the allele is in the range to escape from XCI (DNAme<0.01, blue) or in the range to be subject to XCI (DNAme>0.15, orange). There are mean and median columns for both males and females, but only the female columns are color coded based on XCI status. There are boxes around the genes with female median values with one allele in the range to escape XCI and the other allele in the range to be subject to XCI.

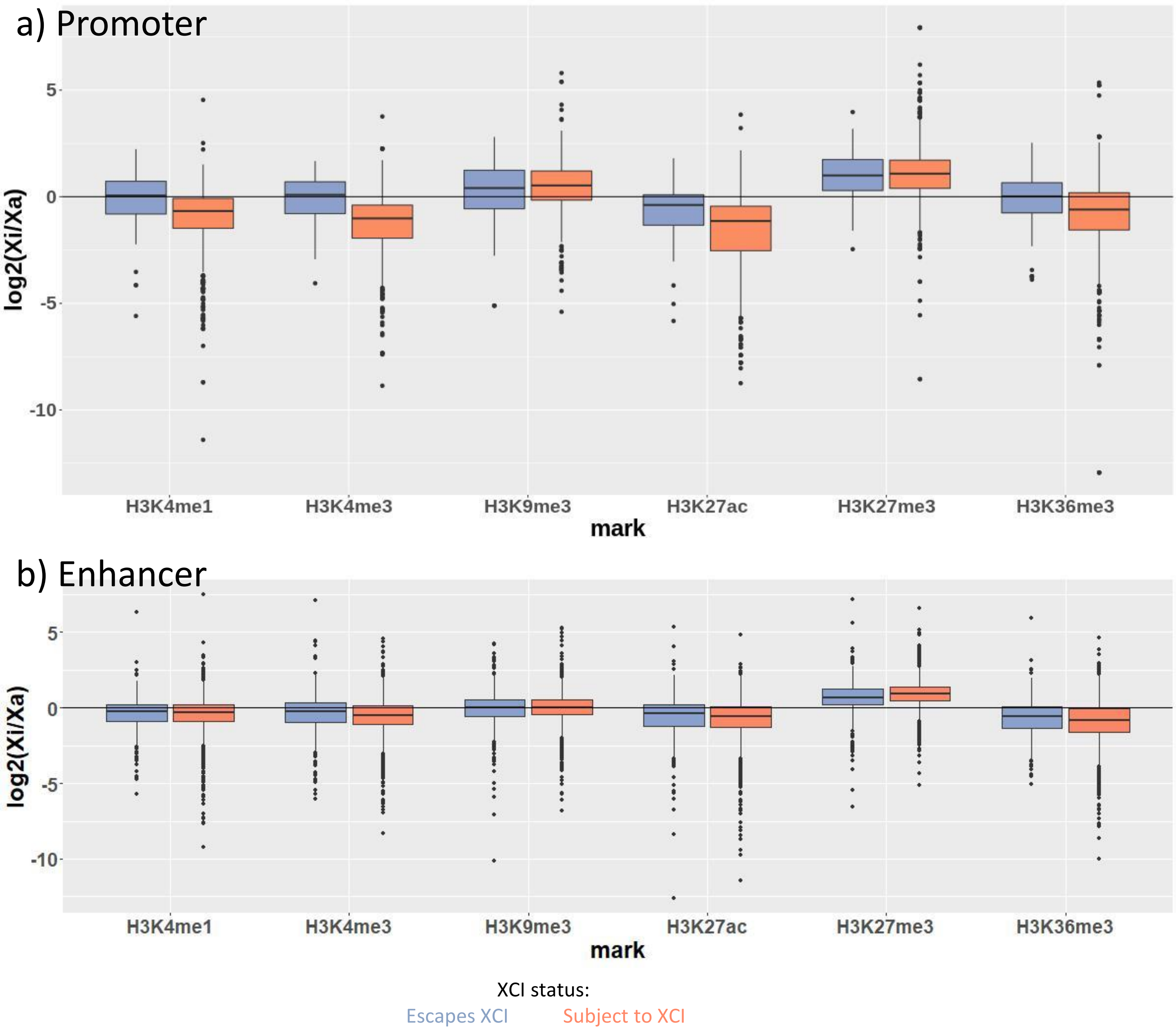

Figure S1: log2(Xi/Xa) for epigenetic marks in CREST. See Table S2 for which comparisons are significant.

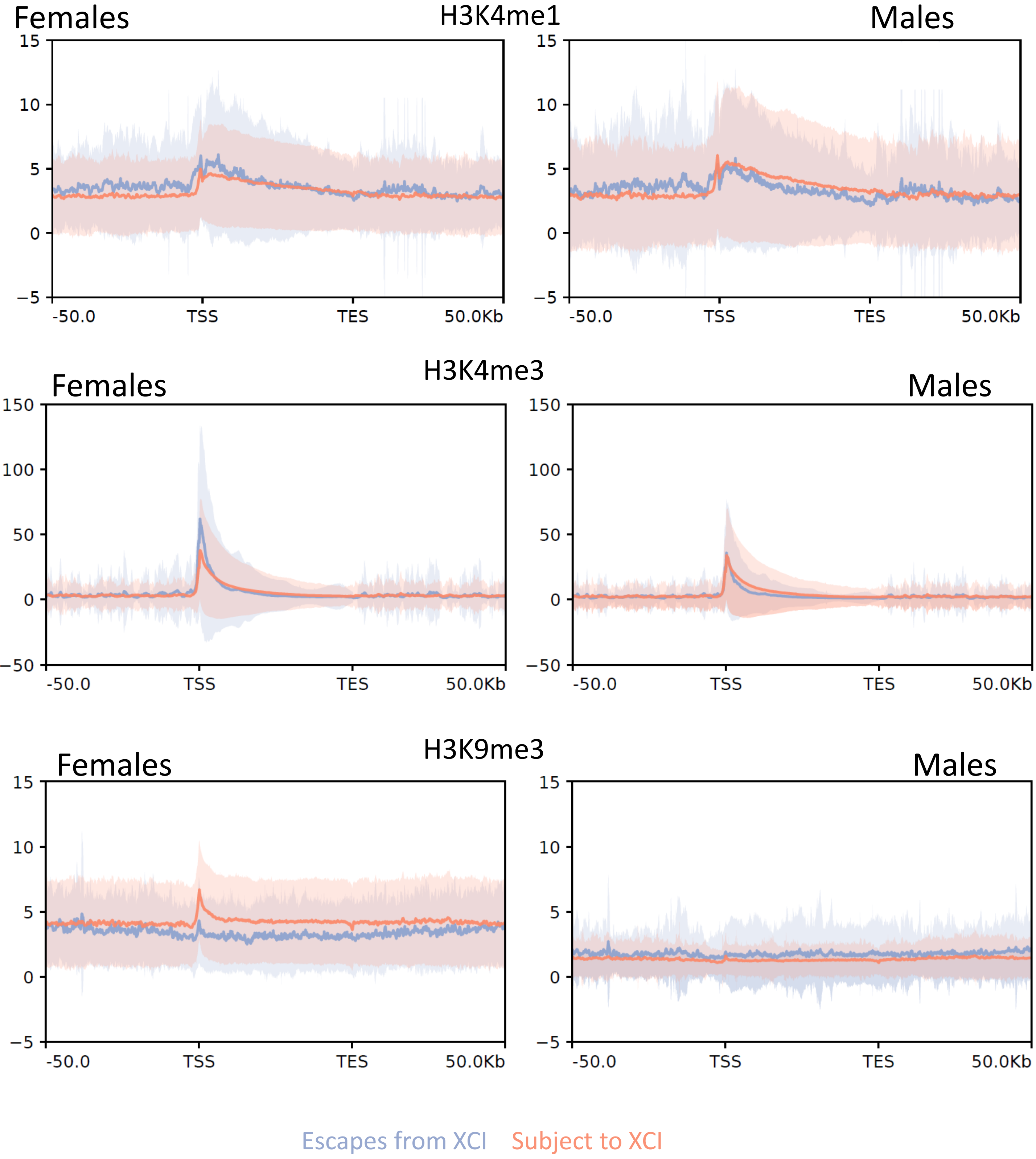

Figure S2: Meta-gene plots of histone marks within 50kb of genes, separated by XCI status. The plots were generated using deeptools computeMatrix and plotProfile on bigwig files that were the mean across samples. Solid lines show the mean values per gene, after having averaged each gene across samples. Lighter shaded regions show the standard deviation of each mark. Continued on next page

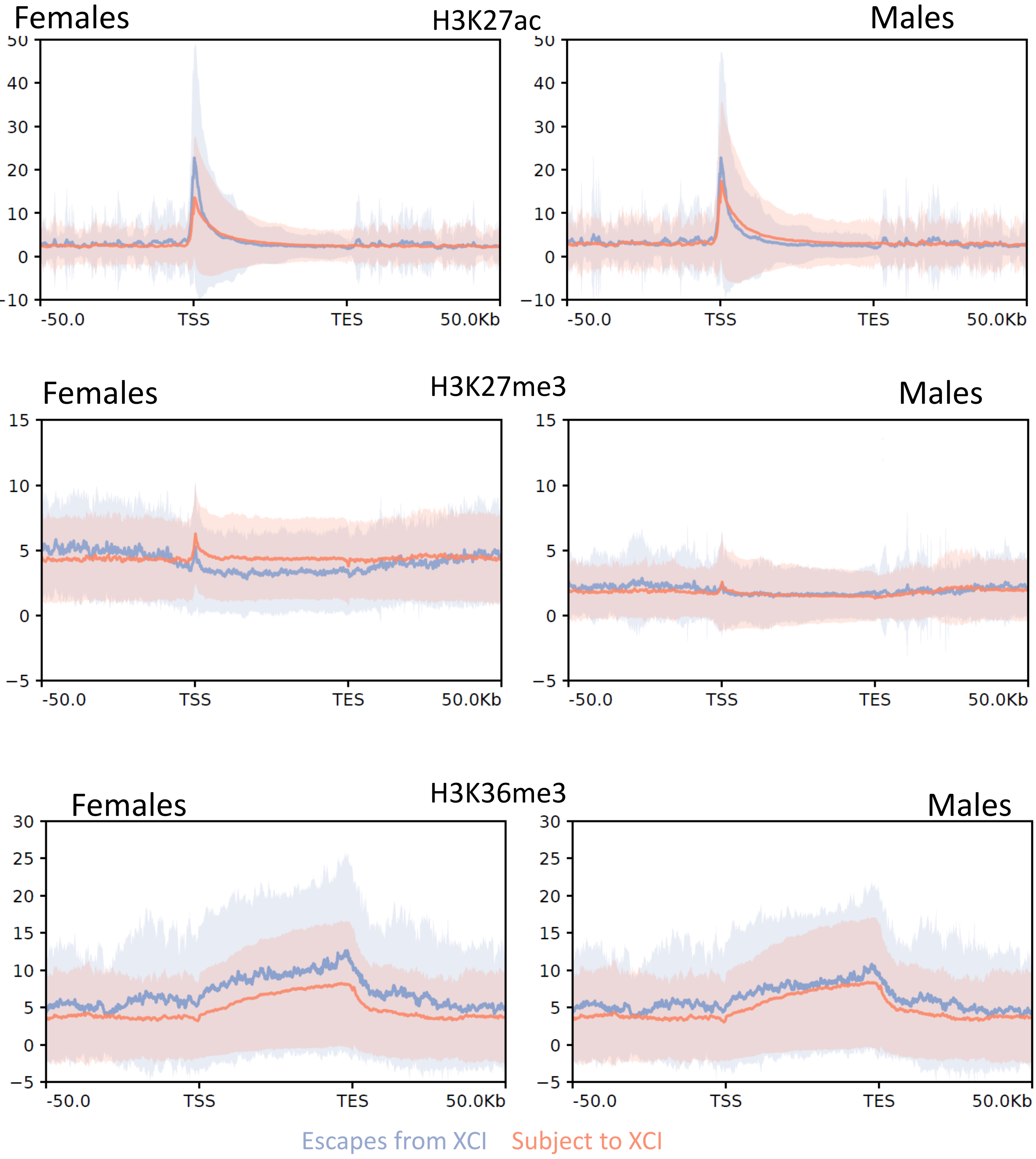

Figure S2: Continued from previous page

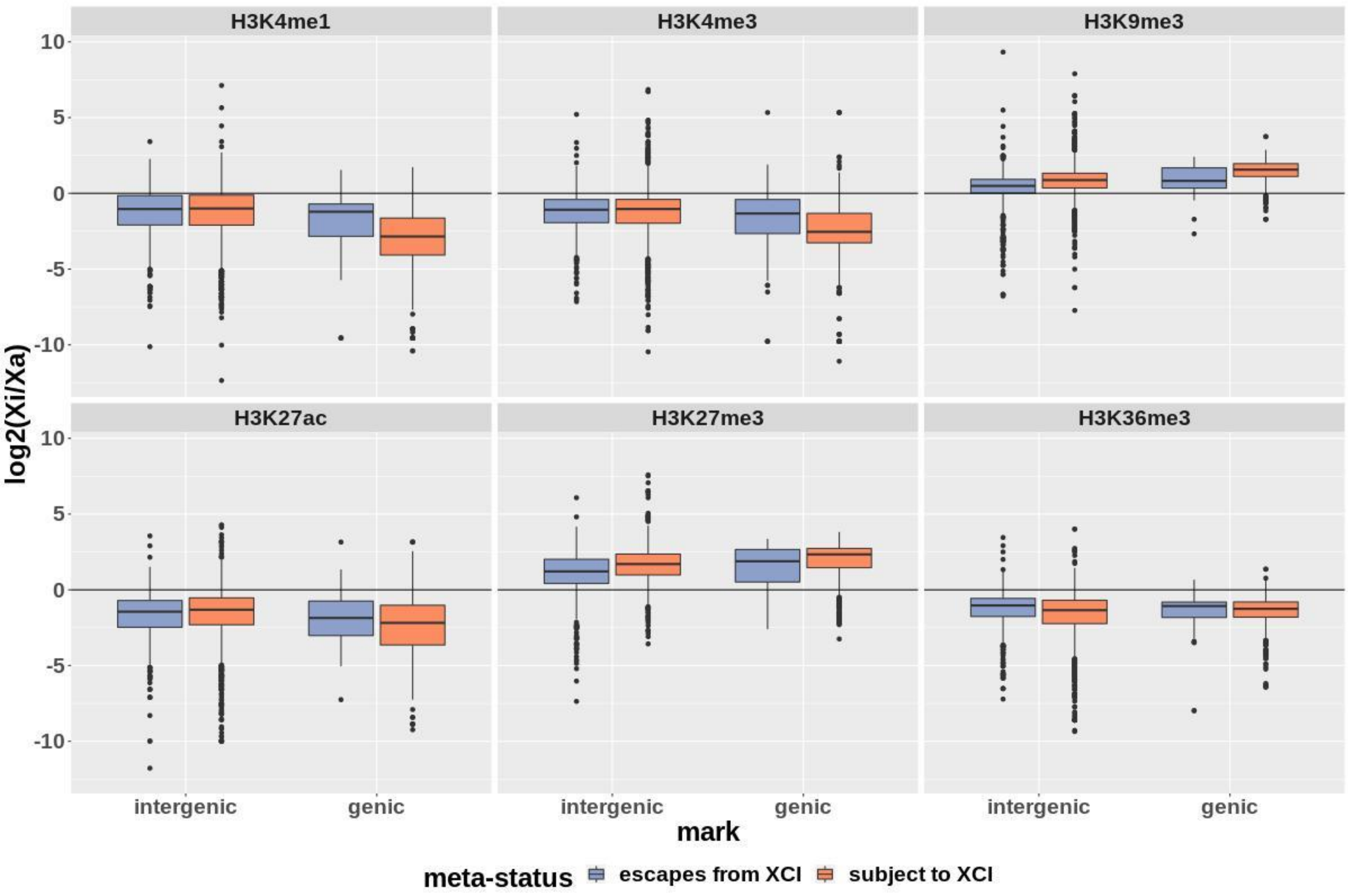

Figure S3: log2(Xi/Xa) for epigenetic marks in CEMT at enhancers mapping to genes which escape from or are subject to XCI. Enhancers are split by whether they are located within a gene (genic) or not (intergenic).

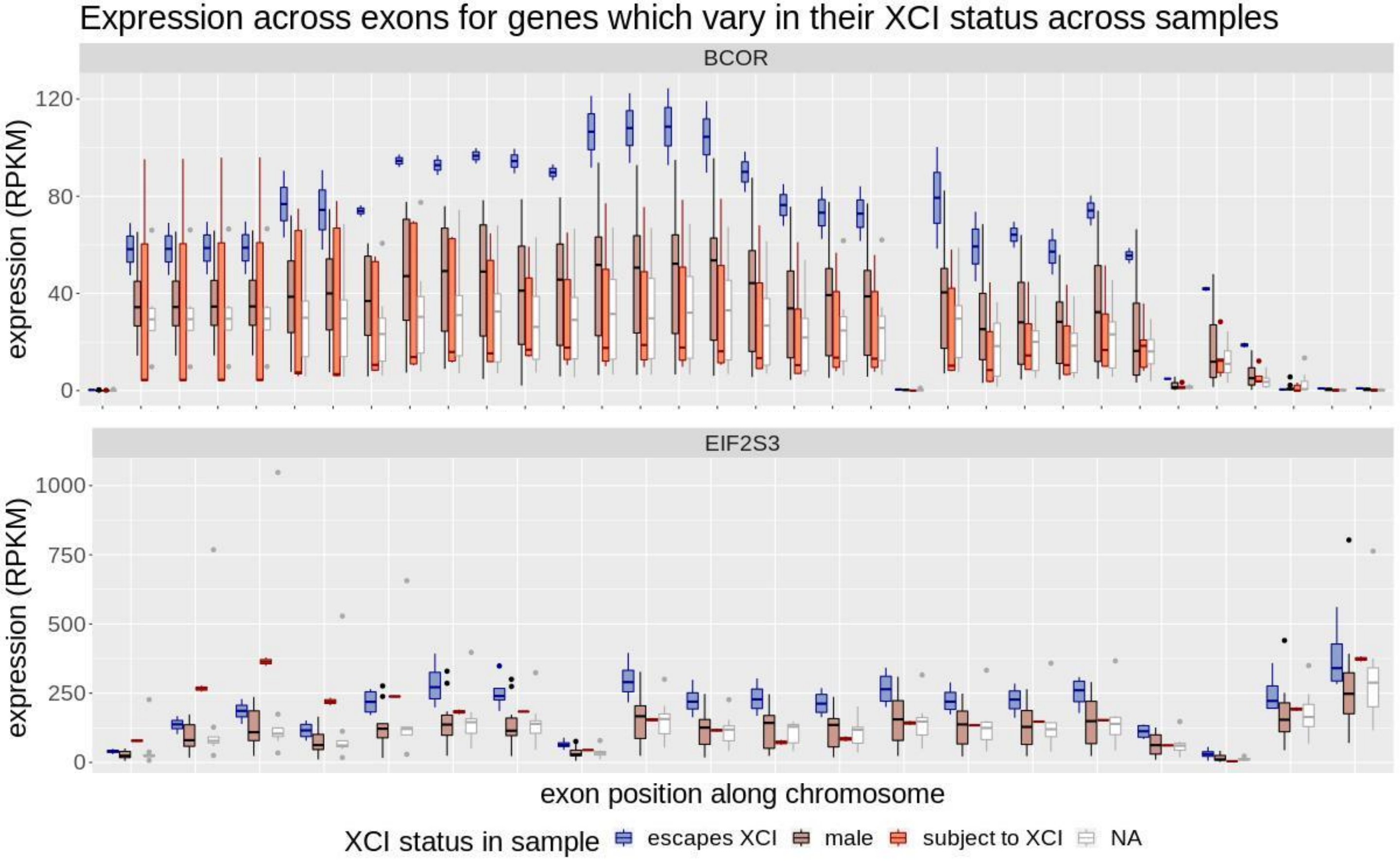

Figure S4: Expression across exons for genes with significantly different expression in samples with opposite XCI statuses. XCI status per sample was determine here using Xi/Xa expression.

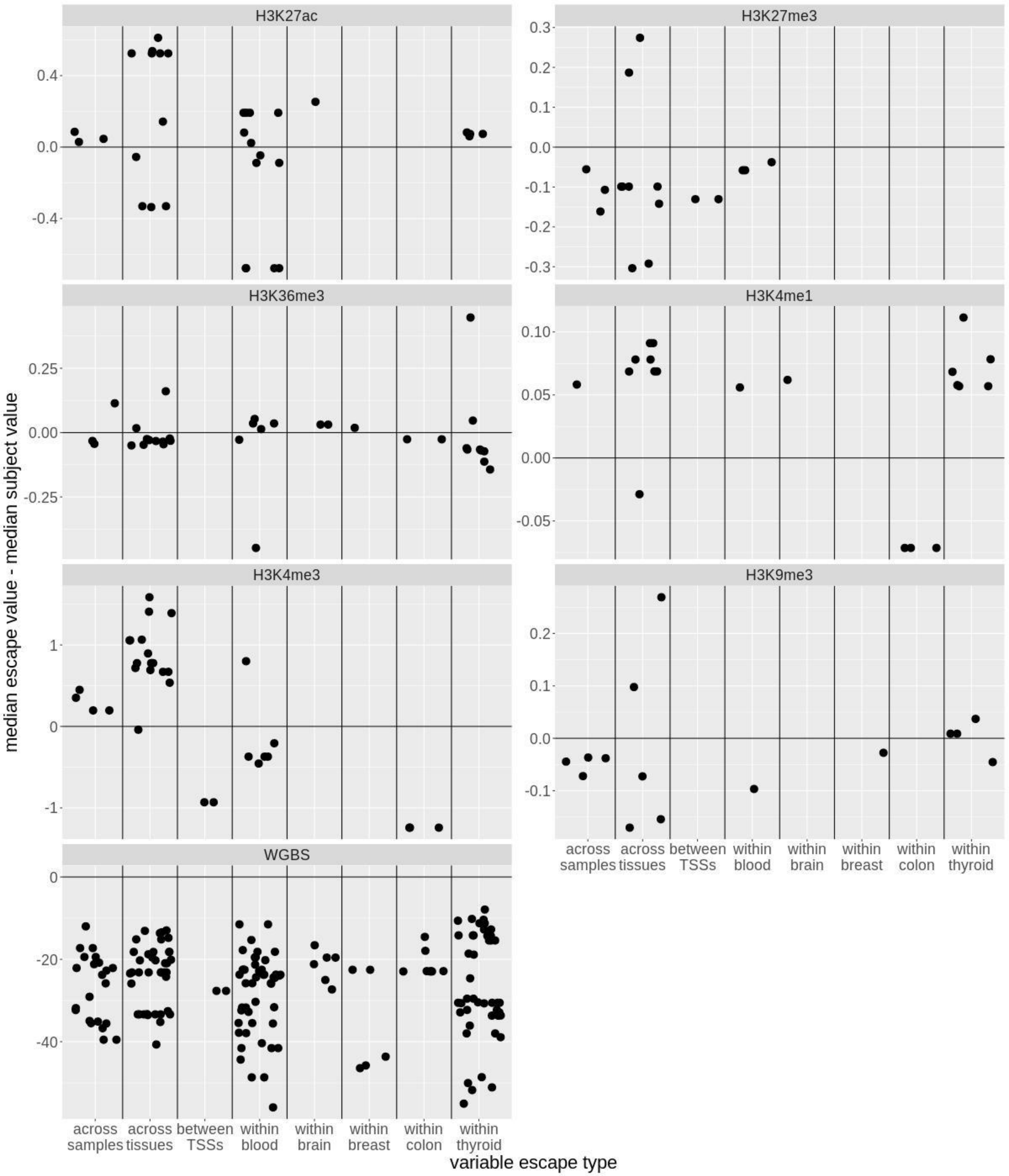

Figure S5: Differences in epigenetic marks between samples found escaping vs subject to XCI at variably escaping genes called using DNAme. For most of these marks, the region 500bp upstream of the promoter is used, except for H3K36me3 which uses the gene body. The median value per gene in samples found subject to XCI was subtracted from the median value per gene in samples which escaped from XCI. This is done here for all genes found variably escaping across individuals by DNAme.

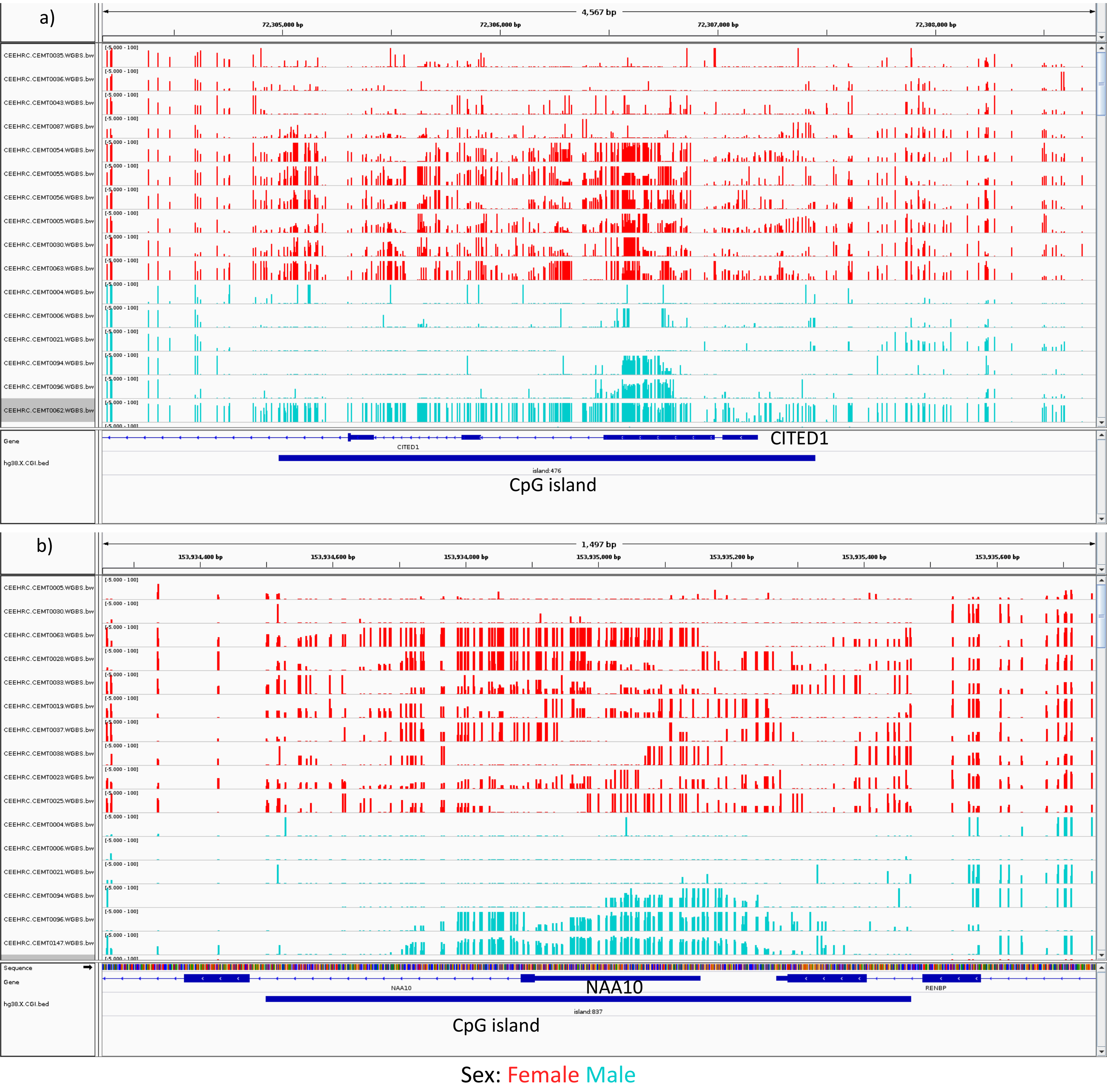

Figure S6: IGV view of DNAme bigwig tracks at two variably escaping genes. a) A view of the CpG island at CITED1. b) a view of the CpG island at NAA10. A broad representation of samples was sought, some hypomethylated, some hypermethylated and some inconsistent across the CpG island. Broad hypermethylation in males at these genes was rare but is included here as an example of an extreme.

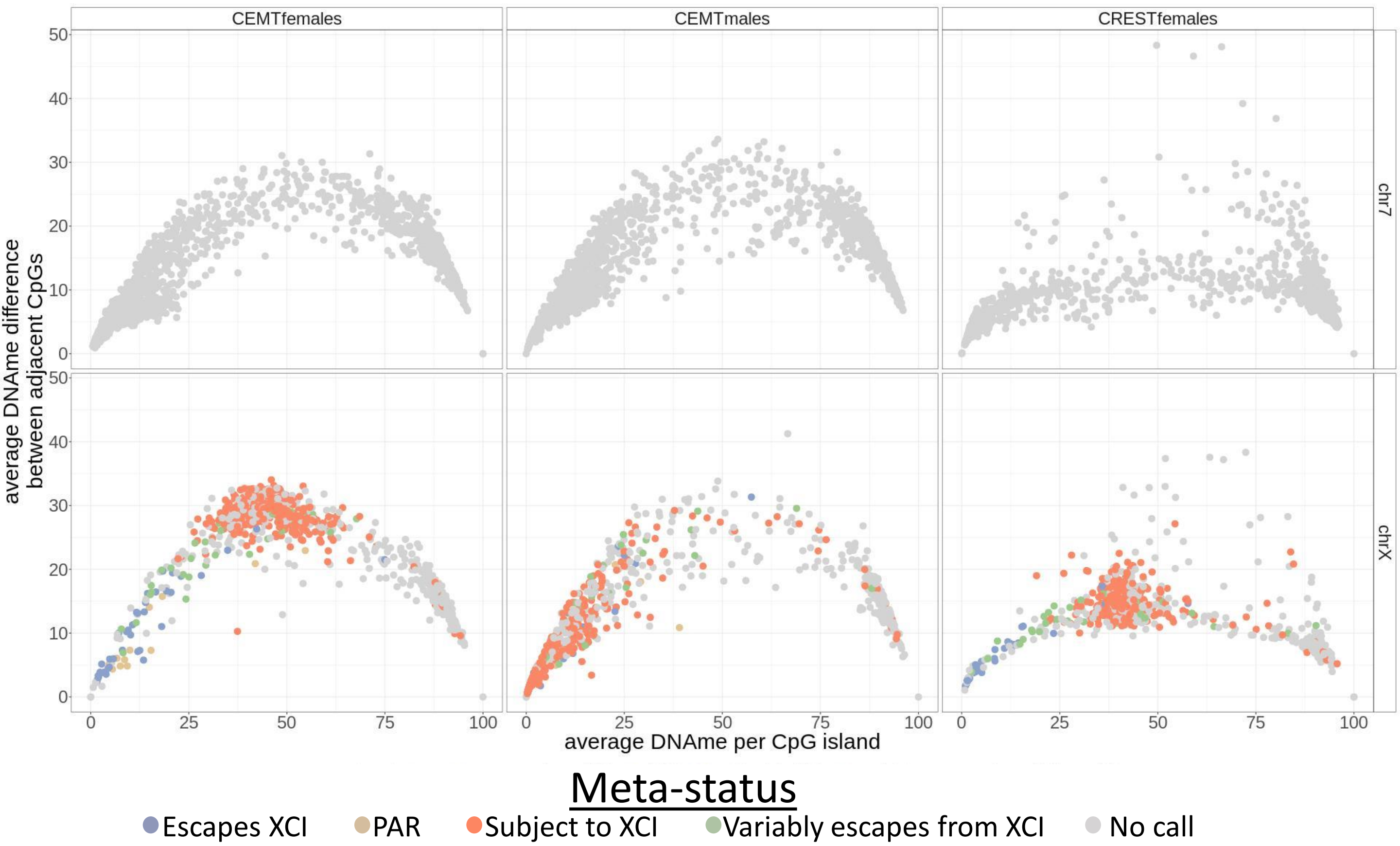

Figure S7: average DNAme difference between adjacent CpGs per CpG island. Each point is the average DNAme difference between adjacent CpGs for an individual island, averaged again across samples. Islands are colored by the meta-status of the closest TSS within 2kb. Chr7 was chosen as an autosomal control to show whether the differences are X specific. Males and females from CEMT were used to check for sex specificity and females from CREST were included to check for cancer specificity.

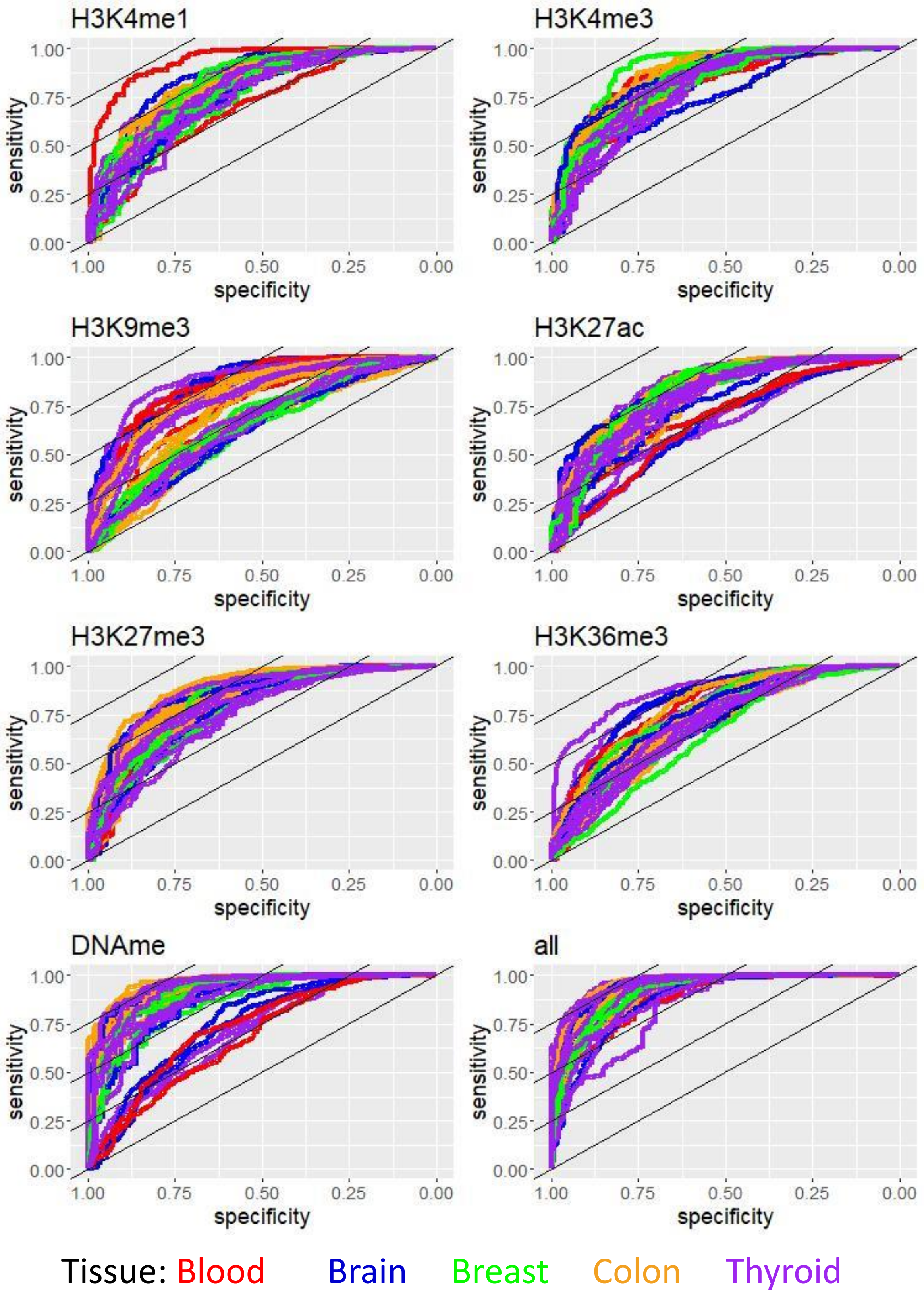

Figure S8: ROC for predictive models trained with each epigenetic mark. On display is one random forest model trained per sample with one epigenetic mark as its input, along with the median value of the mark in similar males. Samples are colored by tissue. The all category is for a predictor using all 6 histone marks and DNAme. Black diagonal lines were added to ease comparison between figures.

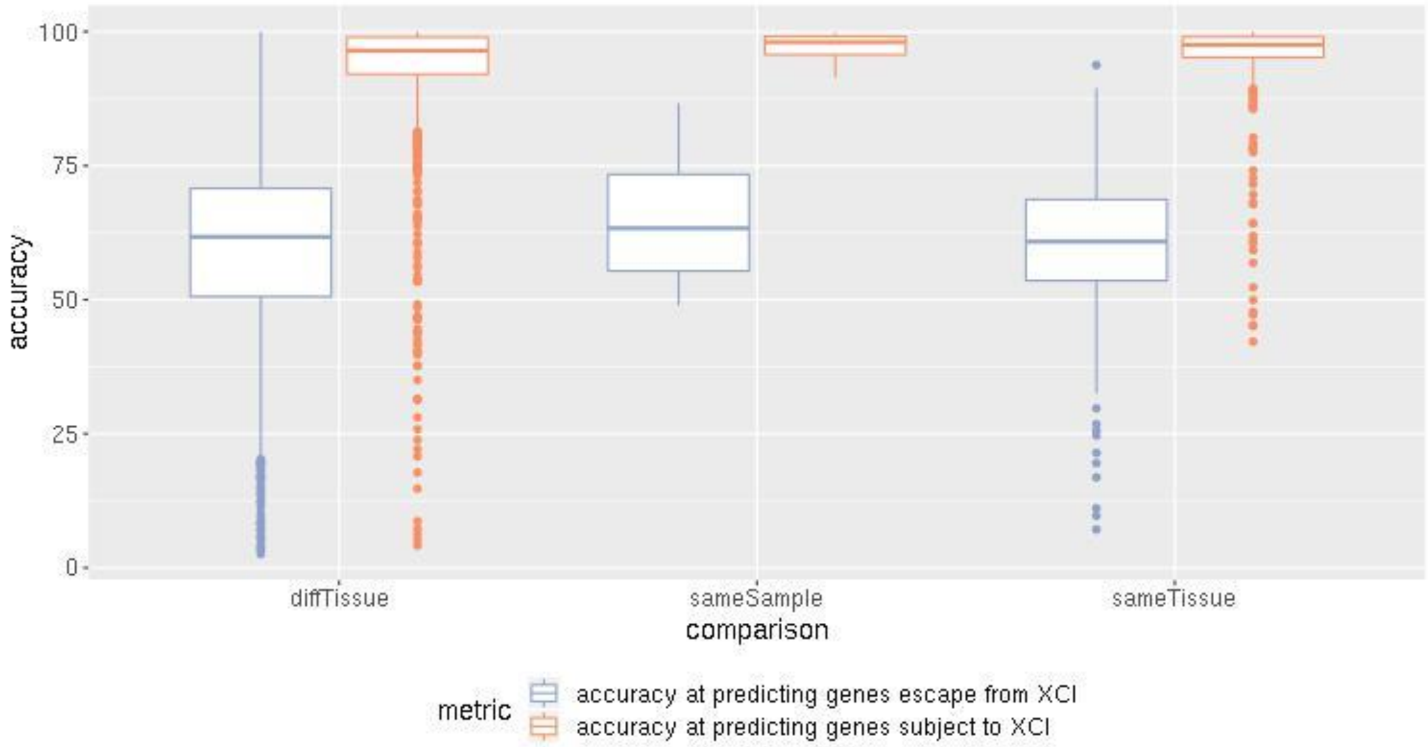

Figure S9: Accuracy when models trained in one sample are tested on other models.

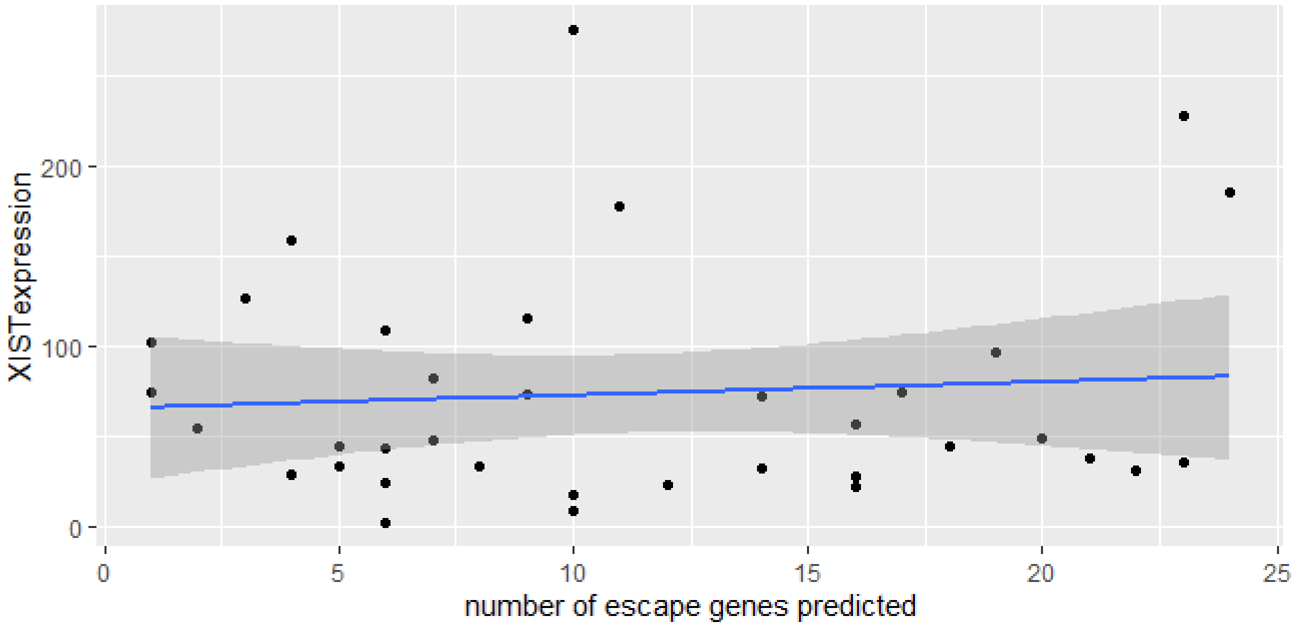

Figure S10: Comparing XIST expression to the number of escape genes predicted per sample. Predictions were made using a random forest model with all histone marks and DNAme.

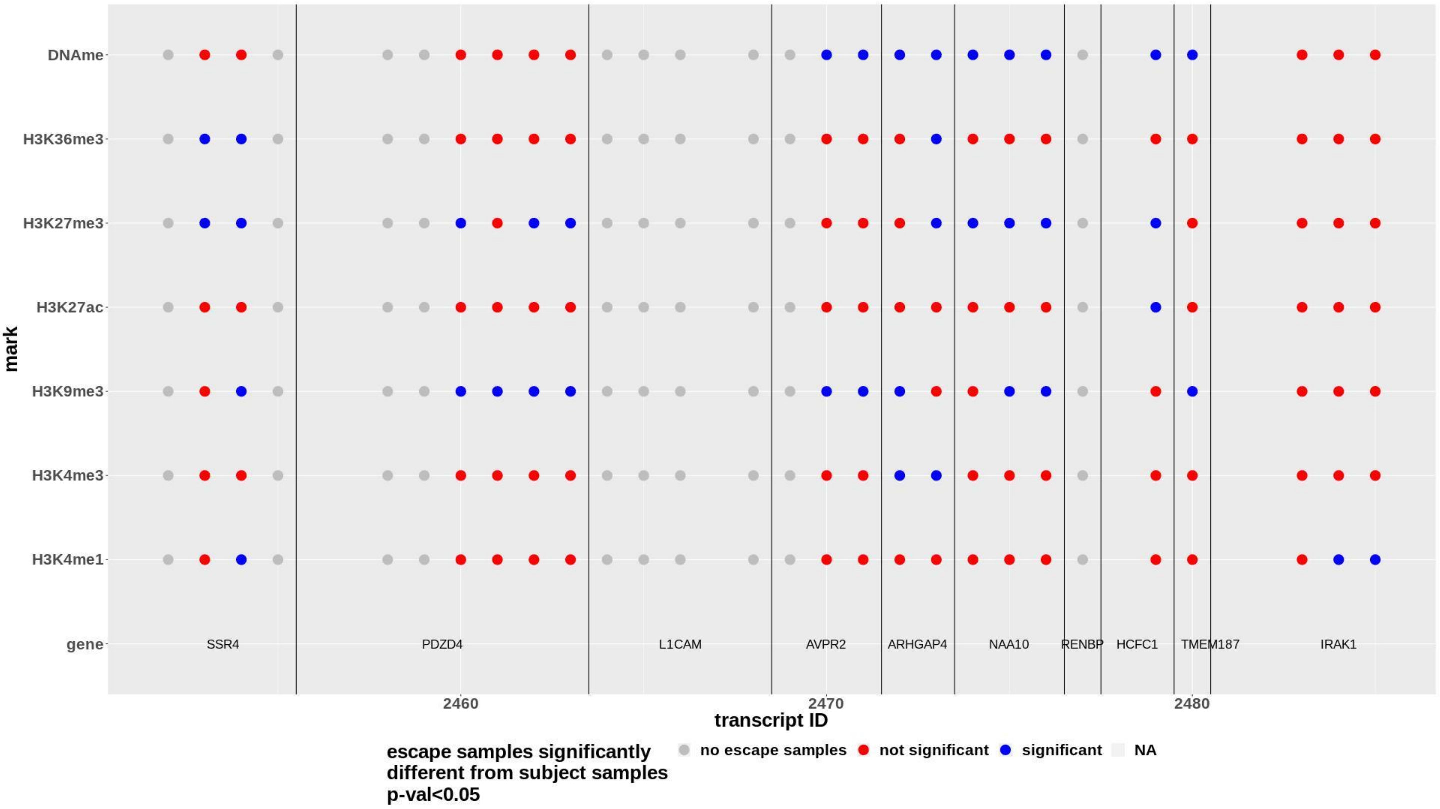

Figure S11: Marks which were significantly different between samples predicted as escaping vs subject to XCI in a variably escaping region. Transcript ID is the order that the transcripts are located along the chromosome. There are multiple transcripts per gene but they may be sharing the same TSS and have the same data for all marks but H3K36me3. Vertical lines are drawn denoting which transcripts belong with each gene.
